# First‐trimester ultrasound detection of fetal heart anomalies: systematic review and meta‐analysis

**DOI:** 10.1002/uog.23740

**Published:** 2022-01-05

**Authors:** J. N. Karim, E. Bradburn, N. Roberts, A. T. Papageorghiou, for the ACCEPTS study, Aris T. Papageorghiou, Zarko Alfirevic, Trish Chudleigh, Hilary Goodman, Christos Ioannou, Heather Longworth, Jehan N. Karim, Kypros H. Nicolaides, Pranav Pandya, Gordon Smith, Basky Thilaganathan, Jim Thornton, Oliver Rivero‐Arias, Helen Campbell, Ed Juszczak, Louise Linsell, Ed Wilson, Lisa Hinton, Jane Fisher, Elizabeth Duff, Anne Rhodes, Gil Yaz

**Affiliations:** ^1^ Nuffield Department of Women's & Reproductive Health University of Oxford Oxford UK; ^2^ Bodleian Health Care Libraries University of Oxford Oxford UK; ^3^ Oxford Maternal & Perinatal Health Institute, Green Templeton College University of Oxford Oxford UK

**Keywords:** cardiac abnormality, congenital heart anomaly, first trimester, positive predictive value, risk, screening, sensitivity, specificity, ultrasound

## Abstract

**Objectives:**

To determine the diagnostic accuracy of ultrasound at 11–14 weeks' gestation in the detection of fetal cardiac abnormalities and to evaluate factors that impact the detection rate.

**Methods:**

This was a systematic review of studies evaluating the diagnostic accuracy of ultrasound in the detection of fetal cardiac anomalies at 11–14 weeks' gestation, performed by two independent reviewers. An electronic search of four databases (MEDLINE, EMBASE, Web of Science Core Collection and The Cochrane Library) was conducted for studies published between January 1998 and July 2020. Prospective and retrospective studies evaluating pregnancies at any prior level of risk and in any healthcare setting were eligible for inclusion. The reference standard used was the detection of a cardiac abnormality on postnatal or postmortem examination. Data were extracted from the included studies to populate 2 × 2 tables. Meta‐analysis was performed using a random‐effects model in order to determine the performance of first‐trimester ultrasound in the detection of major cardiac abnormalities overall and of individual types of cardiac abnormality. Data were analyzed separately for high‐risk and non‐high‐risk populations. Preplanned secondary analyses were conducted in order to assess factors that may impact screening performance, including the imaging protocol used for cardiac assessment (including the use of color‐flow Doppler), ultrasound modality, year of publication and the index of sonographer suspicion at the time of the scan. Risk of bias and quality assessment were undertaken for all included studies using the Quality Assessment of Diagnostic Accuracy Studies (QUADAS‐2) tool.

**Results:**

The electronic search yielded 4108 citations. Following review of titles and abstracts, 223 publications underwent full‐text review, of which 63 studies, reporting on 328 262 fetuses, were selected for inclusion in the meta‐analysis. In the non‐high‐risk population (45 studies, 306 872 fetuses), 1445 major cardiac anomalies were identified (prevalence, 0.41% (95% CI, 0.39–0.43%)). Of these, 767 were detected on first‐trimester ultrasound examination of the heart and 678 were not detected. First‐trimester ultrasound had a pooled sensitivity of 55.80% (95% CI, 45.87–65.50%), specificity of 99.98% (95% CI, 99.97–99.99%) and positive predictive value of 94.85% (95% CI, 91.63–97.32%) in the non‐high‐risk population. The cases diagnosed in the first trimester represented 63.67% (95% CI, 54.35–72.49%) of all antenatally diagnosed major cardiac abnormalities in the non‐high‐risk population. In the high‐risk population (18 studies, 21 390 fetuses), 480 major cardiac anomalies were identified (prevalence, 1.36% (95% CI, 1.20–1.52%)). Of these, 338 were detected on first‐trimester ultrasound examination and 142 were not detected. First‐trimester ultrasound had a pooled sensitivity of 67.74% (95% CI, 55.25–79.06%), specificity of 99.75% (95% CI, 99.47–99.92%) and positive predictive value of 94.22% (95% CI, 90.22–97.22%) in the high‐risk population. The cases diagnosed in the first trimester represented 79.86% (95% CI, 69.89–88.25%) of all antenatally diagnosed major cardiac abnormalities in the high‐risk population. The imaging protocol used for examination was found to have an important impact on screening performance in both populations (*P* < 0.0001), with a significantly higher detection rate observed in studies using at least one outflow‐tract view or color‐flow Doppler imaging (both *P* < 0.0001). Different types of cardiac anomaly were not equally amenable to detection on first‐trimester ultrasound.

**Conclusions:**

First‐trimester ultrasound examination of the fetal heart allows identification of over half of fetuses affected by major cardiac pathology. Future first‐trimester screening programs should follow structured anatomical assessment protocols and consider the introduction of outflow‐tract views and color‐flow Doppler imaging, as this would improve detection rates of fetal cardiac pathology. © 2021 The Authors. *Ultrasound in Obstetrics & Gynecology* published by John Wiley & Sons Ltd on behalf of International Society of Ultrasound in Obstetrics and Gynecology.

## Abstract 2

This article's abstract has been translated into Spanish and Chinese. Follow the links from the abstract to view the translations.

## RESUMEN

**Detección ecográfica de anomalías cardíacas fetales en el primer trimestre: revisión sistemática y metaanálisis**

**Objetivos:**

*Determinar la precisión del diagnóstico mediante ecografía a las 11–14 semanas de gestación en la detección de anomalías cardíacas del feto y evaluar los factores que repercuten en la tasa de detección.*

**Métodos:**

*Este trabajo fue una revisión sistemática de estudios de evaluación de la precisión en el diagnóstico mediante ecografía de la detección de anomalías cardíacas fetales a las 11–14 semanas de gestación, realizada por dos revisores independientes. Se realizó una búsqueda electrónica en cuatro bases de datos (MEDLINE, EMBASE, Web of Science Core Collection y The Cochrane Library) de estudios publicados entre enero de 1998 y julio de 2020. Se incluyeron estudios prospectivos y retrospectivos que evaluaban embarazos con cualquier nivel de riesgo previo y en cualquier entorno sanitario. El estándar de referencia utilizado fue la detección de una anomalía cardíaca en el examen postnatal o póstumo. Se extrajeron los datos de los estudios incluidos con los que se completaron matrices de 2×2. Se realizó un metaanálisis mediante un modelo de efectos aleatorios para determinar la eficacia de la ecografía del primer trimestre en la detección de anomalías cardíacas importantes en general y de los tipos individuales de anomalías cardíacas. Los datos se analizaron por separado para las poblaciones de alto riesgo y las de no alto riesgo. Se realizaron análisis secundarios planificados con anterioridad para evaluar los factores que pueden influir en la eficacia del cribado, como el protocolo de imagen utilizado para la evaluación cardíaca (incluido el uso del Doppler de flujo de color), la modalidad de ecografía, el año de publicación y el índice de sospecha del ecografista en el momento de la prueba. El riesgo de sesgo y la evaluación de la calidad se hicieron para todos los estudios incluidos mediante la herramienta de Evaluación de Calidad de los Estudios de Precisión en el Diagnóstico‐2 (QUADAS‐2, por sus siglas en inglés).*

**Resultados:**

*La búsqueda electrónica produjo 4108 citas. Después de revisar los títulos y los resúmenes, se revisó el texto completo de 223 publicaciones, de las cuales se seleccionaron para su inclusión en el metaanálisis 63 estudios, que informaban sobre 328.262 fetos. En la población de no alto riesgo (45 estudios, 306.872 fetos), se identificaron 1.445 anomalías cardíacas importantes (prevalencia, 0,41% (IC 95%, 0,39–0,43%)). De ellas, 767 se detectaron en la ecografía del corazón en el primer trimestre y 678 no se detectaron. La ecografía del primer trimestre tuvo una sensibilidad combinada del 55,80% (IC 95%, 45,87–65,50%), una especificidad del 99,98% (IC 95%, 99,97–99,99%) y un valor predictivo positivo del 94,85% (IC 95%, 91,63–97,32%) en la población de no alto riesgo. Los casos diagnosticados en el primer trimestre representaron el 63,67% (IC 95%, 54,35–72,49%) de todas las anomalías cardíacas importantes diagnosticadas prenatalmente en la población de no alto riesgo. En la población de alto riesgo (18 estudios, 21.390 fetos), se identificaron 480 anomalías cardíacas importantes (prevalencia, 1,36% (IC 95%, 1,20–1,52%)). De ellas, 338 se detectaron en el examen ecográfico del primer trimestre y 142 no se detectaron. La ecografía del primer trimestre tuvo una sensibilidad combinada del 67,74% (IC 95%, 55,25–79,06%), una especificidad del 99,75% (IC 95%, 99,47–99,92%) y un valor predictivo positivo del 94,22% (IC 95%, 90,22–97,22%) en la población de alto riesgo. Los casos diagnosticados en el primer trimestre representaron el 79,86% (IC 95%, 69,89–88,25%) de todas las anomalías cardíacas importantes diagnosticadas prenatalmente en la población de alto riesgo. El protocolo de imagen utilizado para el examen del feto tuvo una repercusión importante en la eficiencia del cribado en ambas poblaciones (P<0,0001), y se observó una tasa de detección significativamente mayor en los estudios que utilizaron al menos una visualización del infundíbulo ventricular o imágenes Doppler de flujo en color (ambos P<0,0001). Los diferentes tipos de anomalías cardíacas no fueron susceptibles por igual de ser detectados en la ecografía del primer trimestre.*

**Conclusiones:**

*El examen ecográfico del corazón del feto en el primer trimestre permite identificar a más de la mitad de los fetos afectados por una patología cardíaca importante. Los futuros programas de cribado del primer trimestre deberían seguir protocolos de evaluación anatómica estructurados y considerar la introducción de visualizaciones del infundíbulo ventricular y de imágenes Doppler de flujo en color, ya que esto mejoraría las tasas de detección de las patologías cardíacas fetales. © 2021 Los autores. Ultrasound in Obstetrics & Gynecology, publicada por John Wiley & Sons Ltd en nombre de la Sociedad Internacional de Ecografía en Obstetricia y Ginecología.*

## 摘要

**早期妊娠超声检测胎心异常:系统评价和meta分析**

**目的:**

*确定在孕11‐14周超声检测胎儿心脏异常的诊断准确性并评估影响检出率的因素。*

**方法:**

*这是对评估在孕11‐14周超声检测胎儿心脏异常的诊断准确性研究的一项系统评价，由两名独立评审人进行。在四个数据库(美国医学文献联机数据库(MEDLINE)、荷兰医学文摘数据库(EMBASE)、科学引文索引数据库(Web of Science)核心库和考科蓝实证医学资料库(The Cochrane Library))中对1998年1月至2020年7月之间发表的研究进行了一次电子搜索。评估任何先前风险等级下和任何医疗保健背景下的妊娠的前瞻性研究和回顾性研究，都符合纳入本评价的条件。所采用的参考标准是在产后或验尸时检测出心脏异常。用从纳入研究中提取的数据制定出2×2表格。采用随机效应模型进行meta分析，确定在早期妊娠用超声检测大体上重大的心脏异常以及心脏异常的个别类型。将数据按高风险和非高风险人群来单独分析。进行预先计划的二级分析来评估可能影响筛查表现的因素，包括用于心脏评估的影像医疗方案(含彩色多普勒超声的使用)、超声形式、文献发表年代，以及在扫描检查时超声波检查医师的猜测指标。采用诊断准确性研究的质量评价工具(QUADAS‐2)对所含全部研究进行了误差风险和质量评估。*

**结果:**

*电子搜索获得了4108条引文。在对标题和摘要进行回顾之后，对223篇发表文献进行了全文回顾，这其中有63项报告了328262个胎儿的研究被选入进行meta分析。在非高风险群体中(45项研究，306872个胎儿)，识别出1445个重大心脏异常(患病率，0.41%(95% CI，0.39–0.43%))。这其中有767个在早期妊娠超声心脏检查中检出，而有678个未被检出。早期妊娠超声的汇总敏感度为55.80%(95% CI，45.87–65.50%)、特异性为99.98%(95% CI，99.97–99.99%)，以及在非高风险群体中的阳性预测值为94.85%(95% CI，91.63–97.32%)。早期妊娠诊断出的病例在非高风险群体内所有产前诊断出重大心脏异常的病例中占63.67%(95% CI，54.35–72.49%)。在高风险群体中(18项研究，321390个胎儿)，识别出480个重大心脏异常(患病率，1.36%(95% CI，1.20–1.52%))。这其中有338个在早期妊娠超声检查中检出，而有142个未被检出。早期妊娠超声的汇总敏感度为67.74%(95% CI，55.25–79.06%)、特异性为99.75%(95% CI，99.47–99.92%)，以及在非高风险群体中的阳性预测值为94.22%(95% CI，90.22–97.22%)。早期妊娠中诊断出的病例在高风险群体内所有产前诊断出重大心脏异常的病例中占79.86%(95% CI，69.89–88.25%)。发现用于检查的影像医疗方案对两种群体(P<0.0001)的筛查表现有重大影响，在使用了至少一种流出道显示或彩色多普勒超声(均为P<0.0001)的研究中明显观察到更高的检出率。不同类型的心脏异常并不是同样能在早期妊娠超声中检出。*

**结论:**

*早期妊娠超声检查胎心能查出超过半数遭受重大心脏异常的胎儿。未来早期妊娠筛查项目应遵循结构化的解剖学评估方案，并考虑引入流出道显示和彩色多普勒超声，因为这将改胎儿心脏病理的检出率。©2020作者。威利父子公司(John Wiley & Sons Ltd)代表国际妇产科超声学会(ISUOG)出版《国际妇产超声杂志》(Ultrasound in Obstetrics & Gynecology)。*

**CONTRIBUTION**

**What are the novel findings of this work?**

In this systematic review of 63 studies and 328 262 fetuses, first‐trimester ultrasound examination of the fetal heart identified over half of the fetuses affected by major cardiac pathology. There was an independent association between higher detection rate and structured anatomical assessment, with improved screening sensitivity seen when visualization of the outflow tracts and/or color‐flow Doppler imaging were added to the four‐chamber‐view assessment.

**What are the clinical implications of this work?**

When undertaking detailed sonographic examination of the fetal heart in the first trimester, a structured anatomical assessment protocol including visualization of the outflow tracts and the use of color**‐**flow Doppler optimizes the detection of cardiac anomalies.

## INTRODUCTION

Congenital cardiac abnormalities are the most prevalent structural malformation, affecting eight per 1000 fetuses. While the majority of these abnormalities are minor, three per 1000 fetuses suffer from a severe form of cardiac pathology^1^, ^2^. The associated mortality remains high, with recent research linking congenital cardiac abnormalities to over 50% of all infant deaths in England^2^. Importantly, prenatal diagnosis may impact favorably the risk of morbidity and mortality in these neonates^3^, ^4^, ^5^, ^6^, ^7^.

The detection of cardiac abnormalities represents a distinct challenge for prenatal screening, and most occur in patients deemed to be at low *a‐priori* risk^8^, ^9^. In many countries, the gold standard involves second‐trimester evaluation of cardiac anatomy. However, there is widespread variation in how this screening is performed, and detection rates vary owing to different factors, such as the anatomical views obtained routinely and sonographer training^9^, ^10^, ^11^, ^12^. Specialist prenatal echocardiography can diagnose at least 80% of all congenital cardiac abnormalities, but during routine second‐trimester screening, a large proportion of them are still missed^11^.

Reports of successful fetal echocardiography in the first trimester were first described over 30 years ago^13^, ^14^, ^15^, ^16^. Since then, considerable improvements in technology have fueled increasing interest in early anomaly detection^17^, ^18^, ^19^, ^20^. As in the second trimester, routine first‐trimester screening for cardiac anomalies varies between centers and may involve any of the following: assessment without cardiac examination beyond demonstrating a heart beat; routine visualization of the four‐chamber view; detailed examination involving outflow‐tract visualization and Doppler evaluation; or early risk stratification of patients using, for example, nuchal translucency, tricuspid regurgitation or ductus venosus measurements. Thus, there is little international consensus as to how first‐trimester cardiac anatomy assessment should be performed routinely^21^, ^22^, ^23^.

Apart from the value of detecting a cardiac abnormality in itself, the finding is associated independently with fetal aneuploidy, genetic conditions and additional extracardiac malformations^24^, ^25^. Thus, the first‐trimester detection of cardiac abnormalities is complementary to the overarching objective of diagnosing chromosomal abnormalities earlier and will often constitute an indication for invasive prenatal testing rather than screening using cell‐free DNA.

The aim of this study was to determine the diagnostic accuracy of two‐dimensional ultrasound at 11–14 weeks' gestation in the detection of fetal cardiac abnormalities and to evaluate factors that impact the screening performance.

## METHODS

The study protocol for this systematic review was developed and registered with PROSPERO (registration number: CRD42018112434) prior to undertaking the search, selecting the studies and extracting the data. The review of all studies included in the meta‐analysis and the reporting of results were based on the Meta‐Analysis of Observational Studies in Epidemiology (MOOSE), the Synthesizing Evidence from Diagnostic Accuracy Tests (SEDATE) and the Preferred Reporting Items for a Systematic Review and Meta‐Analysis of Diagnostic Test Accuracy Studies (PRISMA‐DTA) guidelines^26^, ^27^, ^28^, ^29^. The Cochrane Collaboration Systematic Reviews of Diagnostic Test Accuracy handbook was also consulted^30^.

The primary outcome was the diagnostic accuracy of two‐dimensional ultrasound at 11–14 weeks' gestation for the detection of major cardiac abnormalities. Secondary outcomes were factors that might impact screening performance (see Statistical Analysis section for details).

### Search strategy

A systematic electronic search strategy was designed with the help of a specialist librarian (N.R.) in order to identify studies evaluating the diagnostic accuracy of two‐dimensional ultrasound in the detection of fetal cardiac abnormalities at 11–14 weeks' gestation (Appendix S1). The search was developed initially using free‐text terms and subject headings related to prenatal screening, early pregnancy and congenital abnormalities, as described previously^19^. In order to increase sensitivity, free‐text terms and subject headings for specific congenital anomalies were incorporated. The search was conducted in MEDLINE (OvidSP), EMBASE (OvidSP), Science Citation Index and Conference Proceedings Citation Index – Science (Web of Science Core Collection) and the Cochrane Database of Systematic Reviews and Cochrane Central Register of Controlled Trials (Cochrane Library, Wiley) from 1 January 1998 to 17 July 2020. Articles written in a language other than English, single‐case reports, commentaries and animal studies were excluded within EndNote X9 (Clarivate, Philadelphia, PA, USA) after full deduplication of references (N.R.).

Study selection was performed in stages by two independent reviewers (J.N.K. and E.B.). Titles and abstracts of citations obtained from the systematic electronic search were reviewed to identify potentially relevant studies. Full texts were subsequently evaluated to determine their eligibility for inclusion. The reference lists of all eligible studies were screened manually for additional citations not identified by the initial electronic search. Agreement regarding inclusion and exclusion of studies was achieved by consensus between the two reviewers or by consultation with a third reviewer (A.T.P.).

### Study selection

Studies reporting on the detection of fetal cardiac abnormalities using two‐dimensional transabdominal (TAS) or transvaginal (TVS) sonography or a combination of both approaches in the first trimester of pregnancy were included. Prospective and retrospective observational studies and randomized controlled trials were eligible for inclusion. Studies evaluating pregnancies with any level of *a‐priori* risk were eligible for inclusion, including those reporting on women with a singleton or multiple pregnancy and in any healthcare setting. Every attempt was made to identify publications from the same research groups that shared screened subjects, and, in such cases, only the study judged to be the most relevant to the aims of the present study or the one with the largest cohort was included. Literature reviews, conference abstracts, case reports with fewer than five subjects, editorials, letters, personal communications and non‐English‐language publications were excluded.

The review included studies that focused exclusively on the first‐trimester ultrasound detection of cardiac abnormalities as well as studies screening for all types of structural fetal abnormality, as long as cardiac abnormalities were included in the reported cohort and an individual breakdown for each cardiac abnormality was reported. Studies that exclusively investigated the use of first‐trimester ultrasound for the detection of fetal chromosomal abnormalities and those that evaluated sonographic markers of cardiac abnormality, such as increased nuchal translucency, tricuspid regurgitation and abnormal ductus venosus flow, were excluded.

Based on previous work of our group^19^, the reported gestational age is often not clearly defined in first‐trimester screening studies, and the gestational age interval of 11–14 weeks could be interpreted as 11 + 0 to 13 + 6, 11 + 0 to 14 + 0 or 11 + 0 to 14 + 6 weeks. In order to ensure a systematic approach, an *a‐priori* decision was made to include all examinations completed within the 14^th^ week up to 14 + 6 weeks' gestation. Prospective studies were included based on their intention to perform screening prior to 14 + 6 weeks, with the understanding that, in real‐life clinical practice, a small proportion of scans may have been performed outside the intended gestational‐age window.

The reference standard for determining the accuracy of first‐trimester cardiac ultrasound assessment was the detection of a cardiac abnormality on postnatal or postmortem examination. Studies that did not state an intention to perform a postnatal or postmortem examination as part of their aims, for the purposes of confirming first‐trimester screening results, were excluded. However, a pragmatic approach was taken: studies that aimed to but did not always achieve complete follow‐up of their patient cohort were still eligible for inclusion in the meta‐analysis. Similarly, postmortem examination was not a requirement for inclusion of individual cases, as this is not always achievable following termination of pregnancy.

### Data extraction

All data included in this review were derived from tables or main text on two independent occasions from each study in order to reduce the risk of error in data collection.

For each study, the following variables were extracted: first author's name, year of publication, sample size, gestational‐age window at the time of screening, population characteristics, study type, patient recruitment details, healthcare setting, index test (i.e. TAS or TVS or both), time allocated to ultrasound assessment, number of sonographers participating in the study and their level of experience, type of cardiac malformations assessed and information regarding postnatal follow‐up. Details regarding the ultrasound protocol used by each study for first‐trimester cardiac assessment were recorded, including evaluation of cardiac situs, cardiac axis, the four‐chamber view, inflow and outflow tracts and the routine use of color‐flow and pulsed‐Doppler techniques.

Data were extracted to populate 2 × 2 tables and to calculate true‐positive, false‐positive, true‐negative and false‐negative rates in order to determine the diagnostic accuracy of first‐trimester ultrasound for the detection of major cardiac abnormalities. The process was repeated to determine the diagnostic accuracy for all types of cardiac abnormality individually, in order to identify those that are most amenable to first‐trimester detection.

Owing to the anticipated heterogeneity of the included studies, considerable effort was made to ensure that the results from the studies were comparable. Thus, data were recorded and analyzed separately for high‐risk populations. High‐risk populations were grouped according to the authors' definition and included patients with a previously affected pregnancy, personal or family history of major cardiac anomaly, pregestational diabetes, increased fetal nuchal translucency, fetal extracardiac abnormalities and multiple pregnancy. Non‐high‐risk populations were defined as a cohort of patients described by the authors as low risk, unselected or mixed risk.

Manual counting of each cardiac abnormality was undertaken and recorded separately from the number of affected fetuses. This was done to enable the assessment of screening characteristics for individual cardiac conditions. For example, if one fetus was affected by atrioventricular septal defect and coarctation of the aorta, we would be able to distinguish between a scenario in which both abnormalities were identified on first‐trimester ultrasound (two true‐positive abnormalities diagnosed; one affected fetus identified correctly in the first trimester) and one in which only the atrioventricular septal defect was identified on first‐trimester ultrasound, with coarctation of the aorta detected only postnatally (one true‐positive diagnosis and one false‐negative diagnosis; one fetus affected by cardiac anomaly identified correctly in the first trimester). The exception to this procedure was in the case of a known cardiac syndrome, such as tetralogy of Fallot, which was considered as one major cardiac anomaly. In addition, a number of studies described the diagnosis of a ‘complex cardiac defect’, which was not defined further, and this was considered as ‘one major cardiac abnormality’ for the purposes of this study.

The commonly used definition of a major cardiac abnormality as being a malformation assumed to be lethal, or requiring surgery or interventional cardiac catheterization during the first year of postnatal life, was followed. Anomalies that are not considered to be structural in nature, but which may require treatment, such as pericardial effusion, hydrops and fetal heart block, were excluded.

### Definition of screen positive

A screen‐positive result following cardiac anatomical ultrasound assessment in the first trimester might reflect one of three possible situations based on the index of suspicion: (1) the diagnosis of a specific cardiac anomaly in the first trimester; (2) the suspicion of a specific cardiac anomaly in the first trimester; or (3) the finding of an anatomical abnormality of undetermined significance (AUS) following assessment of the four‐chamber view or the outflow tracts (e.g. ventricular and/or outflow‐tract disproportion or unclear spatial relationship of the vessels).

All three situations represent a ‘screen‐positive’ test result and, for the primary analysis, detection rates were calculated regardless of the index of suspicion. As different screen‐positive situations may lead to different patient counseling, management and follow‐up strategies, all cardiac anomalies were recorded as diagnosed, suspected or classified as AUS, and true‐positive/false‐positive rates were calculated separately.

We also recognized that a specific diagnostic ‘label’ in the first trimester may be modified later in pregnancy. The anomaly initially identified in the first trimester may evolve (e.g. progression of severe aortic stenosis to hypoplastic left heart syndrome) or may be reclassified (e.g. a ventricular septal defect (VSD) that is subsequently found to be part of tetralogy of Fallot). In this situation, the fetus was identified correctly as having a major cardiac anomaly, but the initial diagnosis was revised. These cases could not be considered fairly as either a true positive or a false positive and were therefore documented separately as ‘a change of first‐trimester diagnosis’.

### Estimation of false‐positive rate and specificity

The false‐positive rate (and therefore specificity) of first‐trimester ultrasound screening is difficult to determine because many fetuses with severe or lethal abnormalities undergo early termination of pregnancy without postmortem confirmation^19^. In order to estimate specificity, reported true‐positive results were assumed to be accurate when they led to termination of pregnancy, even if postmortem confirmation was not available. This is consistent with previous studies in this area, although this practice may lead to under‐ascertainment of the false‐positive rate. In order to address this, a subanalysis of individual fetuses that were assumed to be screen positive and which subsequently received diagnostic confirmation on either postmortem or postnatal examination, was undertaken.

### Quality assessment of studies

Risk of bias and quality assessment were undertaken for all included studies based on the Quality Assessment of Diagnostic Accuracy Studies (QUADAS‐2) tool. This tool evaluates studies within four key domains: patient selection, index test, reference standard and flow of patients through the study. Each study in the review was graded as having either a low, high or unclear risk of bias for each domain and for lack of applicability based on a series of signaling questions developed specifically for this review (Appendix S2).

### Statistical analysis

Meta‐analysis of data extracted from eligible studies was performed in two steps. First, summary statistics with 95% CIs were derived for each study with respect to sensitivity, specificity and positive and negative predictive values of first‐trimester ultrasound anomaly screening for the detection of cardiac pathology per anomaly and per affected fetus. Second, individual study statistics within each population subgroup were combined in order to obtain a pooled summary estimate using a random‐effects model. Haldane–Anscombe correction was used, in which a value of 0.5 was added to cells in 2 × 2 tables, when required, in order to avoid a division‐by‐zero error. Heterogeneity between studies was estimated using the *I* ^2^ statistic.

In the meta‐analysis for the primary outcome, all patients in both population groups with any type of screen‐positive result (diagnosed, suspected or AUS) were included. This allowed us to determine the overall performance of first‐trimester ultrasound in the detection of major cardiac abnormalities in high‐risk and non‐high‐risk populations. For the purposes of the primary analysis, a major cardiac anomaly detected in the first trimester that subsequently changed to a different major cardiac anomaly was considered a true positive.

Preplanned secondary analyses were then conducted to assess factors that might impact the screening performance for major cardiac abnormalities, by determining screening performance in subgroups stratified according to the following: (1) the imaging protocol used for cardiac assessment, such as four‐chamber assessment only, addition of color‐flow Doppler and examination of the outflow tracts; (2) ultrasound modality (TAS *vs* TVS *vs* both); (3) publication year of the study; and (4) the index of diagnostic suspicion (cardiac abnormality diagnosed, suspected or classified as AUS). For all types of cardiac abnormality, a secondary analysis was conducted according to the individual type of cardiac anomaly. For this subanalysis, an *a‐priori* decision was made to perform meta‐analysis only when at least 10 cases of a specific anomaly were present in the pooled sample. Assessment of the impact of gestational age at the time of first‐trimester screening on test sensitivity was planned but not undertaken owing to insufficient data.

Statistical analysis was performed using StatsDirect statistical software version 3.3.0 (StatsDirect Ltd, Altrincham, UK).

## RESULTS

**Figure 1:**
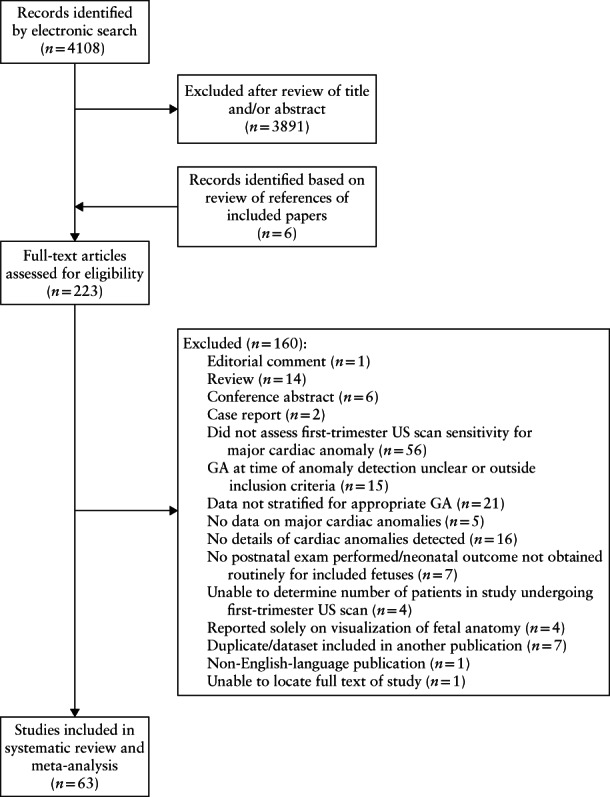
Flowchart summarizing search strategy and study selection in systematic review and meta‐analysis of first‐trimester ultrasound screening for major fetal cardiac abnormalities. GA, gestational age; US, ultrasound.

The electronic search yielded 4108 citations following removal of duplicates, of which 223 underwent full‐text review, resulting in the inclusion of 63 studies^16^, ^17^, ^31^, ^32^, ^33^, ^34^, ^35^, ^36^, ^37^, ^38^, ^39^, ^40^, ^41^, ^42^, ^43^, ^44^, ^45^, ^46^, ^47^, ^48^, ^49^, ^50^, ^51^, ^52^, ^53^, ^54^, ^55^, ^56^, ^57^, ^58^, ^59^, ^60^, ^61^, ^62^, ^63^, ^64^, ^65^, ^66^, ^67^, ^68^, ^69^, ^70^, ^71^, ^72^, ^73^, ^74^, ^75^, ^76^, ^77^, ^78^, ^79^, ^80^, ^81^, ^82^, ^83^, ^84^, ^85^, ^86^, ^87^, ^88^, ^89^, ^90^, ^91^ reporting on 328 262 fetuses in the meta‐analysis (Figure 1). Forty‐five studies^17^, ^31^, ^32^, ^33^, ^34^, ^35^, ^36^, ^37^, ^38^, ^39^, ^40^, ^41^, ^42^, ^43^, ^44^, ^45^, ^46^, ^47^, ^48^, ^49^, ^50^, ^51^, ^52^, ^53^, ^54^, ^55^, ^56^, ^57^, ^58^, ^59^, ^60^, ^61^, ^62^, ^63^, ^64^, ^65^, ^66^, ^67^, ^68^, ^69^, ^70^, ^71^, ^72^, ^73^, ^74^ reported on non‐high‐risk populations (*n* = 306 872 fetuses) (Table S1), while 18 studies^16^, ^75^, ^76^, ^77^, ^78^, ^79^, ^80^, ^81^, ^82^, ^83^, ^84^, ^85^, ^86^, ^87^, ^88^, ^89^, ^90^, ^91^ assessed high‐risk women (*n* = 21 390 fetuses) (Table S2).

**Figure 2:**
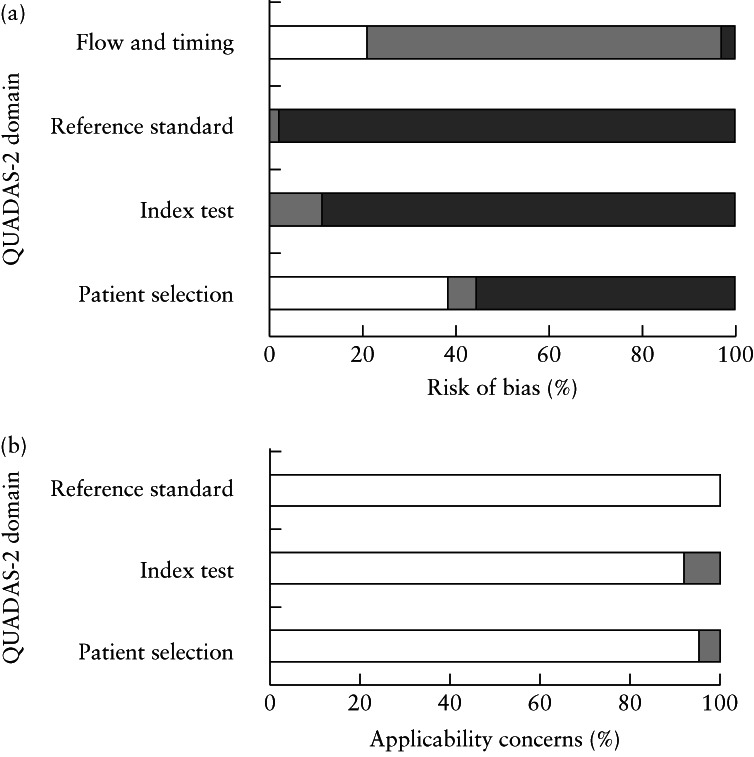
Quality assessment of studies included in systematic review, for risk of bias (a) and study applicability (b), based on QUADAS‐2 guidance. 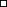, low; 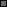, high; 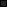, unclear.

The included studies were published between 1998 and 2020. Studies were performed in a variety of healthcare settings, although the majority (*n* = 49) took place, at least in part, in either a university hospital or a tertiary‐care affiliated center^16^, ^17^, ^31^, ^32^, ^33^, ^34^, ^36^, ^38^, ^40^, ^42^, ^43^, ^46^, ^47^, ^49^, ^50^, ^52^, ^55^, ^56^, ^57^, ^58^, ^59^, ^60^, ^61^, ^62^, ^63^, ^64^, ^65^, ^66^, ^67^, ^68^, ^69^, ^71^, ^72^, ^73^, ^74^, ^75^, ^76^, ^77^, ^78^, ^79^, ^81^, ^84^, ^85^, ^86^, ^87^, ^88^, ^89^, ^90^, ^91^ (Tables S1 and S2). Five studies performed multicenter data collection. The methodological quality assessment of the included studies is summarized in Figure 2, and details of the imaging protocols of each study are summarized in Tables S3 and S4.

### Screening performance for major cardiac abnormalities

#### Non‐high‐risk population

**Figure 3:**
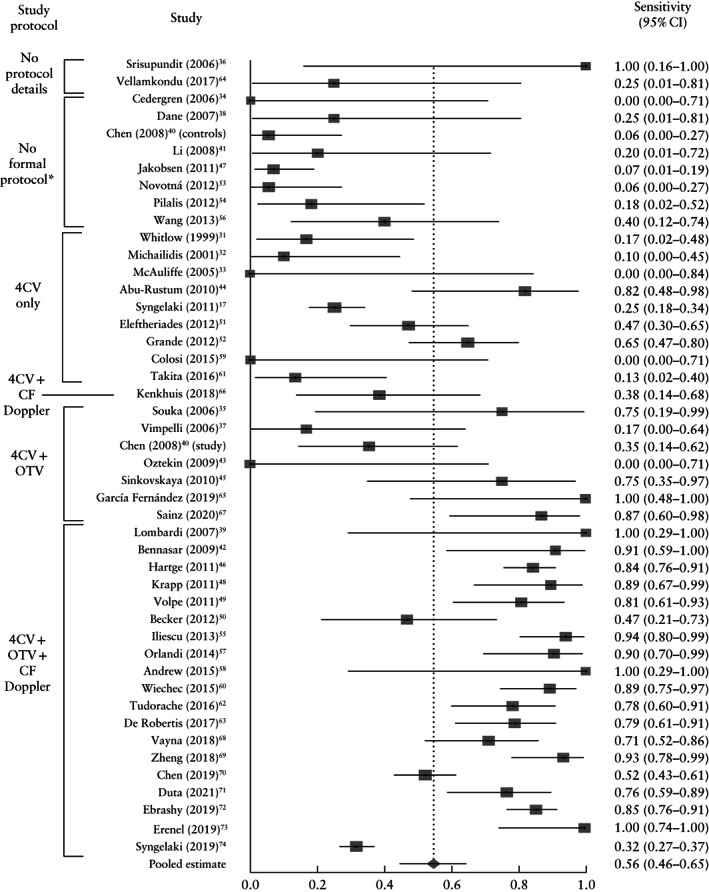
Forest plot of sensitivity of first‐trimester ultrasound in the detection of major fetal cardiac abnormalities in non‐high‐risk populations, which included low‐risk, mixed‐risk and unselected populations. Only first author of each study is given. *I* ^2^ = 91.8% (95% CI, 90.3–93.0%). *‘No formal protocol’ was defined as absence of a dedicated ultrasound checklist or a protocol without a dedicated cardiac assessment. 4CV, four‐chamber view; CF, color flow; OTV, outflow‐tract view.

In the non‐high‐risk population, a total of 306 872 fetuses were screened and 1445 major cardiac anomalies were identified, yielding a prevalence of major cardiac anomaly of 0.41% (fixed‐effects model, 95% CI, 0.39–0.43%). Of these, 767 were detected on first‐trimester ultrasound, while the remaining 678 were not detected; a further 43 cases were false positive. Based on the pooled analysis, first‐trimester ultrasound screening had a sensitivity of 55.80% (95% CI, 45.87–65.50%), specificity of 99.98% (95% CI, 99.97–99.99%) and positive predictive value of 94.85% (95% CI, 91.63–97.32%) (Table 1 and Figure 3). Abnormalities diagnosed in the first trimester represented 63.67% (95% CI, 54.35–72.49%) of all antenatally diagnosed major cardiac abnormalities (Table 1).

**Table 1:** Screening performance of first‐trimester ultrasound imaging in the detection of major fetal cardiac abnormalities in non‐high‐risk and high‐risk populations

Parameter	Non‐high risk	High risk
Fetuses screened	306 872	21 390
Studies included	45	18
Total number of major cardiac abnormalities (TP + FN)	1445	480
TP	767	338
Sensitivity	55.80 (45.87–65.50)	67.74 (55.25–79.06)
Specificity	99.98 (99.97–99.99)	99.75 (99.47–99.92)
Positive predictive value	94.85 (91.63–97.32)	94.22 (90.22–97.22)
Proportion of all antenatally detected major cardiac abnormalities*	63.67 (54.35–72.49)	79.86 (69.89–88.25)

Data are given as *n* or % (95% CI).

Values reflect global detection rate calculated and refer to any screen‐positive result following cardiac anatomical assessment in the first trimester based on the index of suspicion: diagnosis of a specific major cardiac abnormality, suspicion of a specific major cardiac abnormality or detection of an abnormality of unknown significance in the four‐chamber or outflow‐tract view.

* Proportion of all major cardiac abnormalities identified antenatally (i.e. excluding anomalies detected postnatally) detected on first‐trimester ultrasound.

FN, false negative; TP, true positive.

On analysis per fetus (26 studies, 99 621 fetuses), 340/585 fetuses with a major cardiac abnormality were identified on first‐trimester ultrasound (pooled sensitivity, 63.78% (95% CI, 51.21–75.45%); pooled specificity, 99.98% (95% CI, 99.97–99.99%))^17^, ^31^, ^33^, ^34^, ^37^, ^38^, ^39^, ^41^, ^42^, ^43^, ^45^, ^46^, ^48^, ^49^, ^51^, ^55^, ^56^, ^57^, ^60^, ^62^, ^63^, ^64^, ^65^, ^67^, ^69^, ^73^.

Of the 699 major cardiac anomalies that were diagnosed (*n* = 683) or suspected (*n* = 16) on first‐trimester ultrasound and assumed to be true positive, 155 (22.17%) were confirmed by postmortem or postnatal examination (Table S5).

#### High‐risk population

**Figure 4:**
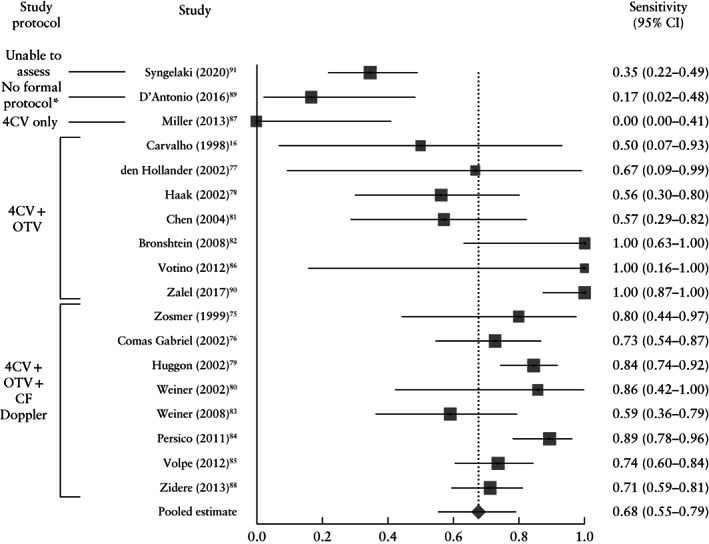
Forest plot of sensitivity of first‐trimester ultrasound in the detection of major fetal cardiac abnormalities in high‐risk populations. Only first author of each study is given. *I* ^2^ = 85.8% (95% CI, 79.1–89.6%). *‘No formal protocol’ was defined as absence of a dedicated ultrasound checklist or a protocol without a dedicated cardiac assessment. 4CV, four‐chamber view; CF, color flow; OTV, outflow‐tract view.

In the high‐risk population, a total of 21 390 fetuses were screened and 480 major cardiac anomalies were identified, yielding a prevalence of major cardiac anomaly of 1.36% (fixed‐effects model, 95% CI, 1.20–1.52%). Of these, 338 were detected on first‐trimester ultrasound, while the remaining 142 were not detected; a further 20 cases were false positive. Based on the pooled analysis, first‐trimester ultrasound screening had a sensitivity of 67.74% (95% CI, 55.25–79.06%), specificity of 99.75% (95% CI, 99.47–99.92%) and positive predictive value of 94.22% (95% CI, 90.22–97.22%) (Table 1 and Figure 4). Abnormalities diagnosed in the first trimester represented 79.86% (95% CI, 69.89–88.25%) of all antenatally diagnosed major cardiac abnormalities (Table 1).

On analysis per fetus (14 studies, 6854 fetuses), 180/241 fetuses with a major cardiac abnormality were identified on first‐trimester ultrasound (pooled sensitivity, 70.00% (95% CI, 55.65–82.59%); pooled specificity, 99.61% (95% CI, 99.16–99.89%))^16^, ^75^, ^76^, ^77^, ^78^, ^81^, ^82^, ^83^, ^85^, ^86^, ^87^, ^88^, ^89^, ^90^.

Of the 335 major cardiac anomalies that were diagnosed (*n* = 320) or suspected (*n* = 15) on first‐trimester ultrasound and assumed to be true positive, 73 (21.79%) were confirmed by postmortem or postnatal examination (Table S6).

### Factors affecting screening performance

#### Imaging protocol

Studies were classified into five subgroups, according to the imaging protocol used: (1) systematic protocol not reported; (2) assessment of the four‐chamber view without color‐flow Doppler; (3) assessment of the four‐chamber view with color‐flow Doppler; (4) assessment of the four‐chamber view and at least one outflow‐tract view without color‐flow Doppler; and (5) assessment of the four‐chamber view and at least one outflow‐tract view with color‐flow Doppler examination (Tables S3 and S4).

Analysis of these protocol subgroups demonstrated significant differences in sensitivity on pairwise comparisons using χ^2^ and linear trend testing in both the non‐high‐risk and high‐risk populations (all *P* < 0.0001) (Tables 2 and S7). This analysis showed an increase in first‐trimester screening sensitivity with increasing level of detail of the anatomical protocol used.

**Table 2:** Impact of imaging protocol on the sensitivity of first‐trimester ultrasound in the detection of major fetal cardiac anomalies in non‐high‐risk populations

Parameter	Anatomical protocol				
	No formal protocol*	4CV only	4CV + CF Doppler	4CV + OTV	4CV + OTV + CF Doppler
Studies	8	9	1	7	19
Fetuses	35 121	85 287	5534	8033	171 860
Pooled sensitivity	13.51 (7.05–21.67)	32.96 (18.18–49.71)	38.46 (13.86–68.42)	57.54 (31.41–81.58)	80.04 (67.94–89.84)

Data are given as *n* or % (95% CI).

χ^2^ test (2 by k) comparing the five protocol types showed a significant difference in their sensitivity (*P* < 0.0001), while χ^2^ test for linear trend suggested a statistically significant increase in screening sensitivity with increasing level of detail of the imaging protocol used (*P* < 0.0001).

* ‘No formal protocol’ was defined as absence of a dedicated ultrasound checklist or a protocol without a dedicated cardiac assessment.

This table includes only studies with protocols available for analysis (Table S3).

The protocol was not available in two studies^36^, ^64^.

One study^40^ included both a control group (no formal protocol) and a study group (4CV + OTV).

4CV, four‐chamber view; CF, color flow; OTV, outflow‐tract view.

We assessed imaging factors that could affect the detection rate of routine ultrasound screening in the non‐high‐risk group. Evaluation of at least one outflow‐tract view and the use of color‐flow Doppler in addition to the four‐chamber view assessment were associated independently with a significantly higher rate of detection (both *P* < 0.0001) (Table 3). This analysis was not undertaken in high‐risk cases, as targeted ultrasound meant that almost all fetuses in this cohort were evaluated using an extended imaging protocol that included assessment of the outflow tracts.

**Table 3:** Impact of color‐flow (CF) Doppler and outflow‐tract view (OTV) on the sensitivity of first‐trimester ultrasound in the detection of major fetal cardiac anomalies in non‐high‐risk populations

Parameter	Additional value of CF Doppler			Additional value of OTV		
	Without CF Doppler	With CF Doppler	*P*	Without OTV	With OTV	*P* *
Studies	16	20	—	10	26	—
Fetuses	93 320	177 394	—	90 821	179 893	—
Pooled sensitivity	42.49 (28.41–57.24)	78.38 (66.39–88.32)	< 0.0001	33.79 (20.12–49.00)	75.37 (64.31–84.95)	< 0.0001

Data are given as *n* or % (95% CI).

* χ^2^ test (2 by k).

#### Ultrasound mode

Evaluation of the impact of mode of ultrasound was also performed in the non‐high‐risk group. The vast majority of studies used both TAS and TVS (*n* = 36; 294 185 fetuses), while a minority of studies used solely TAS (*n* = 9; 17 444 fetuses) or TVS (*n* = 2; 648 fetuses). χ^2^ test (2 by k) showed no statistical difference when comparing detection rate between the three modalities (*P* = 0.4662) (Table 4).

**Table 4:** Impact of ultrasound mode on the sensitivity of first‐trimester ultrasound in the detection of major fetal cardiac anomalies in non‐high‐risk populations

Parameter	Ultrasound mode		
	TAS only	TVS only	TAS and TVS
Studies	8	2	34
Fetuses	16 296	648	279 634
Pooled sensitivity	56.54 (33.85–77.88)	57.06 (1.76–99.99)	55.43 (43.37–67.16)

Data are given as *n* or % (95% CI).

χ^2^ test (2 by k) showed no significant difference between the three approaches (*P* = 0.423).

Details regarding the mode of ultrasound used were not available in one study^70^.

TAS, transabdominal sonography; TVS, transvaginal sonography.

#### Publication year

Analysis by year of study publication (in or before 2004, 2005–2009, 2010–2014 or in or after 2015) in the non‐high‐risk population demonstrated improved screening sensitivity with more recent year of publication (*P* = 0.0006), but no such trend was seen in the high‐risk group.

#### Diagnostic certainty

The screening performance of first‐trimester ultrasound examination according to diagnostic certainty is shown in Table 5. In the non‐high‐risk population, there were 767 anomalies detected on ultrasound, of which 683 were given a diagnosis, 16 were suspected and 68 were considered AUS. Among the cases given a label (diagnosed or suspected) in the non‐high‐risk group, 10 had a change of diagnosis. Detailed information on the non‐high‐risk group is provided in Tables S8–S11. In the high‐risk population, there were 338 anomalies detected on ultrasound, of which 320 were given a diagnosis, 15 were suspected and three were considered AUS. Among the cases given a label (diagnosed or suspected), 19 had a change of diagnosis. Detailed information on the high‐risk group is provided in Tables S12–S15.

**Table 5:** Screening performance of first‐trimester ultrasound in the detection of major fetal cardiac anomalies, according to diagnostic certainty, in non‐high‐risk and high‐risk populations

Parameter	Index of suspicion			
	Major cardiac anomaly diagnosed (Analysis 1)	Major cardiac anomaly suspected (Analysis 2)	AUS in 4CV and/or OTV (Analysis 3)	Studies screening exclusively for AUS in 4CV and/or OTV (Analysis 4)*
Non‐high‐risk population				
Studies evaluated	42	9	1	3
Fetuses evaluated	299 075	34 125	5534	7997
Screen positive†	698	36	1	75
True positive	674	15	0	68
Change of diagnosis	9	1	—	—
False positive	15	20	1	7
Pooled sensitivity‡	51.20 (40.92–61.43)	44.60 (15.08–76.41)	0.00 (0.00–36.94)	83.10 (74.30–90.35)
Pooled specificity	99.99 (99.99–100.00)	99.96 (99.88–100.00)	99.98 (99.90–100.00)	99.90 (99.81–99.96)
Pooled PPV	96.58 (93.95–98.48)	67.81 (27.84–96.37)	0.00 (0.00–97.50)	91.27 (71.81–99.84)
High‐risk population				
Studies evaluated	18	6	4	—
Fetuses evaluated	21 342	3547	1205	—
Screen positive†	326	27	5	—
True positive	304	12	3	—
Change of diagnosis	16	3	—	—
False positive	6	12	2	
Pooled sensitivity‡	65.27 (52.31–77.17)	24.43 (13.21–37.79)	13.37 (0.01–37.37)	—
Pooled specificity	99.93 (99.84–99.98)	99.28 (98.17–99.88)	99.73 (99.07–100.00)	—
Pooled PPV	97.65 (95.76–98.99)	60.73 (40.41–79.29)	55.79 (12.91–93.81)	—

Data are given as *n* or % (95% CI).

This table provides a breakdown of screen‐positive results obtained by first‐trimester ultrasound screening according to index of suspicion of the sonographer: (1) diagnosis of a specific major cardiac anomaly in the first trimester; (2) suspicion of a specific major cardiac anomaly in the first trimester; or (3) finding of an abnormality of unknown significance (AUS) in either the four‐chamber (4CV) or outflow‐tract (OTV) view.

* Studies^44^, ^60^, ^63^ in Analysis 4 screened exclusively for abnormalities in the 4CV or OTVs (e.g. ventricular and/or outflow‐tract disproportion, abnormality of spatial relationship of vessels) with the objective of providing a formal and specific diagnosis at a more advanced gestational age.

Therefore, these three studies were excluded from Analyses 1, 2 and 3.

† Number of anomalies identified in the first trimester refers to all screen‐positive anomalies that were diagnosed, suspected or labeled as AUS, which included true‐positive and false‐positive diagnoses and cases in which the initial first‐trimester diagnosis was subsequently changed.

‡ For calculation of sensitivity for diagnosis of major cardiac anomaly, a false‐negative case was defined as any anomaly that was not diagnosed, suspected or labeled as AUS in the first trimester in each study.

Similarly, for calculation of sensitivity for suspected major cardiac anomaly in the first trimester, a false‐negative case was defined as any anomaly that was not diagnosed, suspected or labeled as AUS in the first trimester in each study.

PPV, positive predictive value.

#### Screening for individual cardiac anomalies

The screening performance of first‐trimester ultrasound for individual types of cardiac anomaly that affected at least 10 cases was assessed in both high‐ and non‐high‐risk groups. In the non‐high‐risk group, cardiac anomalies were grouped into those with a detection rate of > 60%, 25–60% or < 25% (Tables 6 and S16). The 12 individual types of cardiac anomaly that affected more than 10 cases in the high‐risk population are reported in Table S17. Differences in detection rates between non‐high‐risk and high‐risk women are reported in Table S18. In both non‐high‐risk and high‐risk populations, VSD was associated with a higher rate of a false‐positive finding and change of diagnosis compared with other anomalies assessed in the study (Tables S16 and S17).

**Table 6:** Screening performance of first‐trimester ultrasound in the detection of individual types of fetal cardiac anomaly in non‐high‐risk population

Anomaly	Sensitivity (% (95% CI))
Detection rate > 60%	
Ectopia cordis	93.26 (76.03–99.98)
Hypoplastic right heart syndrome	91.65 (77.23–99.21)
Tricuspid atresia/dysplasia	88.63 (76.00–96.94)
Atrioventricular septal defect	77.24 (63.62–88.42)
Truncus arteriosus	76.73 (58.94–90.62)
Complex cardiac defect	76.31 (57.46–90.92)
Hypoplastic left heart syndrome	73.28 (59.86–84.82)
Heterotaxy syndrome	72.59 (55.75–86.63)
Single ventricle	71.21 (52.11–87.03)
Double‐outlet right ventricle	63.11 (44.90–79.59)
Detection rate of 25–60%	
Pulmonary atresia	59.68 (23.63–90.53)
Transposition of the great arteries	45.05 (29.29–61.35)
Tetralogy of Fallot	40.95 (30.16–52.20)
Aortic valve stenosis	38.81 (15.77–64.90)
Coarctation of the aorta	37.23 (23.96–51.56)
Ebstein's anomaly	25.03 (4.83–54.08)
Detection rate < 25%	
Ventricular septal defect	23.92 (14.41–34.97)
Atrial septal defect	21.53 (6.78–41.66)
Pulmonary valve or artery stenosis	19.45 (8.99–32.74)
Rhabdomyoma	4.87 (0.19–22.09)

## DISCUSSION

In this meta‐analysis including 328 262 screened fetuses, we show, firstly, that the majority of cardiac anomalies can be identified at the 11–14‐week scan, secondly, that imaging protocols have an important impact on screening performance, with a significantly higher detection rate observed in studies using outflow‐tract views and color‐flow Doppler imaging, and thirdly, that the type of cardiac anomaly under evaluation has a strong impact on detection rate.

In non‐high‐risk populations, which were unselected or had an *a‐priori* low or mixed risk, first‐trimester ultrasound assessment identified just over half (56%) of major cardiac abnormalities, which constituted approximately two‐thirds (64%) of all major cardiac anomalies detected antenatally. In the high‐risk population, the detection rate was higher, with over two‐thirds (68%) of cases detected on first‐trimester ultrasound, representing approximately 80% of all major cardiac abnormalities detected antenatally. The positive predictive value of an abnormal first‐trimester cardiac assessment was approximately 95% in both groups (Table 1).

The finding of a higher detection rate for cardiac abnormalities in high‐risk, compared to lower‐risk, populations is in keeping with findings of previous studies on first‐trimester fetal anomaly detection^18^, ^19^ and is probably due to targeted screening: increased awareness when the *a‐priori* risk is high will result in a more detailed examination to provide early reassurance or confer high‐risk status.

### Clinical implications

After a first‐trimester cardiac evaluation, possible outcomes are: (1) the diagnosis of a major cardiac anomaly; (2) the suspicion of a major cardiac anomaly; (3) an anatomical variant of undetermined significance; (4) an inconclusive result secondary to inadequate imaging; and (5) early reassurance in the context of normal findings. Many studies have concentrated on treating the scan as a diagnostic test. In our analysis, we evaluated the diagnostic accuracy of the scan as a screening test, considering women in categories 1–3 described above as screen positive, those in category 5 as screen negative and those in category 4 as ‘no‐call’. We believe that greater clarity in future reporting will better inform future screening strategies.

If we are to screen in the first trimester, how should this be done? Directly relevant is the finding that use of an anatomical protocol is associated with increased detection of fetal cardiac abnormalities. A ‘dose–response’ improvement in the detection rate with increasing detail of the anatomical study protocol was seen in all population groups (Tables 2 and S7). The strength of this association, clinical plausibility and similar findings from previous studies further support the notion that this is not a chance finding^19^, ^92^, ^93^.

Our data suggest that, when undertaking routine screening for fetal cardiac anomaly at 11–14 weeks, an outflow‐tract view and color‐flow Doppler should be included, as both have a statistically significant impact on the detection rate (Table 3). Studies using the most extensive cardiac protocols (four‐chamber view with outflow‐tract view and color‐flow Doppler) reported detection rates in the non‐high‐risk population that were comparable with those in the high‐risk population (Tables 2 and S7).

Barriers to implementation of such protocols include the high level of sonographer training required as well as appropriate allocation of time and the use of high‐resolution ultrasound equipment. It is likely that the combined impact of these factors contributed to the overall increased detection rates seen in studies with more detailed protocols, although it was not possible to examine this given the limitations of the data. Another consideration is the safety of Doppler before 14 weeks^21^, ^94^, although color‐flow Doppler is considered safe at 11–14 weeks as long as the ALARA (as low as reasonably achievable) principle is followed^23^, ^95^, ^96^. Studies assessing the use of Doppler during first‐trimester cardiac screening have demonstrated that this assessment is consistently feasible with a thermal index (TI) and mechanical index (MI) well below the maximum levels recommended for practice and that a satisfactory assessment is possible within 3–4 min of exposure time, not only for experienced sonographers but also through the learning curve^97^, ^98^, ^99^. Finding the balance between (demonstrated) benefits of improved diagnostic accuracy and (theoretical) risk needs to be considered when undertaking screening.

There is no consensus on whether TAS or TVS should be used for primary screening^18^, ^100^. This analysis did not demonstrate a difference in screening performance for cardiac anomalies when comparing TAS alone, TVS alone and a combination of the two (Table 4). However, very few studies relied on a single ultrasound modality, with the majority of studies using a combination of both TAS and TVS, most commonly beginning with TAS followed by TVS when visualization with the former was inadequate. We believe that the choice of ultrasound modality will continue to be tailored to patient preference, clinician expertise and other factors, such as obesity^101^.

### Detection of individual cardiac anomalies

It was possible to categorize cardiac abnormalities based on our ability to detect them in the first trimester on ultrasound (Tables 6, S16 and S17). The variation seen is logical: for some anomalies, for example stenotic valvular pathologies or narrowing of the pulmonary artery and aortic arch, pathophysiological mechanisms involve gradual changes *in utero*, meaning that such abnormalities may be amenable to diagnosis only at a more advanced gestational age or even postnatally^11^, ^102^. For other anomalies, such as VSD, their size may be below the resolution of ultrasound imaging. It is therefore unlikely that first‐trimester ultrasound will ever be able to detect every fetus affected by these types of abnormality. We should acknowledge that the focus of first‐trimester screening should be primarily on the detection of anomalies that might impact prenatal decision‐making and care, as patients affected by these anomalies are those who will benefit most from an early diagnosis. Our review has shown that a comprehensive first‐trimester cardiac evaluation can detect a very high proportion of certain cardiac anomalies, including complex cardiac defects, single‐ventricle pathology, ectopia cordis, heterotaxy, atrioventricular septal defect and valvular atresia.

### Strengths and limitations

In this systematic review, we have assessed the totality of the existing evidence regarding the diagnostic accuracy of first‐trimester ultrasound screening for fetal cardiac anomalies. The study was undertaken using a prospective and registered protocol and involved detailed extraction of individual data on cardiac anomalies. Preplanned subgroup analyses based on the *a‐priori* risk of the population group, index of suspicion at the time of scan, anatomical protocol and mode of ultrasound allowed an in‐depth understanding of first‐trimester cardiac screening and yielded evidence‐based recommendations for future work.

Our study has some expected limitations. Many of the studies analyzed as part of this systematic review were performed in centers of excellence and often by a small group of highly experienced experts (Tables S1 and S2). There may also be an element of reporting bias from authors wishing to demonstrate positive results. As a consequence, pooled first‐trimester detection rates in this review are comparable with (if not higher than) those reported from second‐trimester cardiac screening initiatives. This means that our findings reflect the highest standards available in our field, which may not be achievable on a larger scale^2^, ^11^, ^103^, ^104^, ^105^, ^106^. Useful data in this regard come from one of the largest multicenter studies, involving 476 sonographers, which may provide a more realistic estimate of what can be achieved by a high‐quality, first‐trimester population‐based cardiac screening program^74^ (Table S8). In addition, considerable heterogeneity between the included studies was observed. This was mitigated by subgroup analysis and strict definitions regarding the types of cardiac anomaly included in the analysis. Variation remains among studies in their inclusion and exclusion criteria, sonographer experience, level of detail of postnatal examination, length of postnatal follow‐up and outcome reporting. Variation also exists in the nomenclature of cardiac anomalies, as defined by individual study authors: for example, hypoplastic right heart syndrome, tricuspid atresia, pulmonary atresia with intact septum and univentricular heart may all be overlapping diagnoses. However, we believe that this is a secondary issue, as the detection of a cardiac abnormality is more important than the precise anatomical diagnosis. Despite the limitations described above, we believe that the pooled data provided us with the best estimate of first‐trimester ultrasound screening performance and the factors that affect it.

An important challenge faced in this study was the determination of false‐positive rates. As with other major anomalies in the first trimester, early surgical termination may preclude postmortem examination. In this study, we found that only approximately 22% of all assumed true‐positive results had a reported physical secondary confirmation (Tables S5 and S6), resulting in relative uncertainty regarding the exact false‐positive rate of first‐trimester cardiac ultrasound evaluation. We attempted to quantify this uncertainty by assessing each individual first‐trimester cardiac diagnosis in relation to secondary confirmation. A large proportion of false‐positive cases were cases with low diagnostic certainty (i.e. suspected and AUS cases) (Table 5). Our best estimate is that the false‐positive rate is low: in the most relevant group (non‐high‐risk group), there were 674 true‐positive diagnoses, nine changes of diagnosis and 15 reported false‐positive diagnoses. Therefore, only 15/698 (2.1%) diagnoses were false positive. This low rate is reassuring, but we call on researchers to report reference tests (postmortem, subsequent imaging or postnatal examination) clearly and comprehensively in future screening studies, including a clear statement of the proportion of cases in which this was not available.

### Conclusions

This study provides strong evidence that first‐trimester examination of the fetal heart allows effective stratification by identifying a cohort of fetuses at high risk of a cardiac anomaly. Based on the available data and uncertainty regarding false‐positive rates, the action after a positive screening scan should be expert fetal cardiac ultrasound follow‐up. The development of information and support for parents will also be a key consideration. Future first‐trimester screening programs should follow a standard anatomical assessment protocol and recognize that not all anomalies are amenable to detection and that some evolve during pregnancy based on their natural history. Combined with appropriate training and implementation of referral pathways, this would be expected to have an important positive impact on the earlier detection of fetal cardiac anomalies.

## Supporting information


**Appendix S1** Search strategy
**Appendix S2** QUADAS‐2 tool
**Appendix S3** Members of the Assessing Clinical and Cost Effectiveness of Prenatal first‐Trimester anomaly Screening (ACCEPTS) study group

**Table S1** Characteristics of studies reporting on the detection of major cardiac anomalies by first‐trimester ultrasound in non‐high‐risk populations
**Table S2** Characteristics of studies reporting on the detection of major cardiac anomalies by first‐trimester ultrasound in high‐risk populations
**Table S3** Details of anatomical protocols used by studies evaluating non‐high‐risk populations
**Table S4** Details of anatomical protocols used by studies evaluating high‐risk populations
**Table S5** Number of major cardiac anomalies diagnosed or suspected in the first trimester with independent secondary confirmation, in non‐high‐risk populations
**Table S6** Number of major cardiac anomalies diagnosed or suspected in the first trimester with independent secondary confirmation, in high‐risk populations
**Table S7** Impact of first‐trimester imaging protocol on the detection of major cardiac anomalies in high‐risk populations
**Table S8** Characteristics of major cardiac anomalies diagnosed following first‐trimester ultrasound assessment in non‐high‐risk populations
**Table S9** Characteristics of major cardiac anomalies suspected following first‐trimester ultrasound assessment in non‐high‐risk populations
**Table S10** Characteristics of major cardiac anomalies reported as cardiac abnormality of unknown significance (AUS) following first‐trimester ultrasound assessment in non‐high‐risk populations
**Table S11** Details of cases in which diagnosis or suspicion of a specific major cardiac anomaly made in the first trimester was changed, in non‐high‐risk populations
**Table S12** Characteristics of major cardiac anomalies diagnosed following first‐trimester ultrasound assessment in high‐risk populations
**Table S13** Characteristics of major cardiac anomalies suspected following first‐trimester ultrasound assessment in high‐risk populations
**Table S14** Characteristics of major cardiac anomalies reported as cardiac abnormality of unknown significance (AUS) following first‐trimester ultrasound assessment in high‐risk populations
**Table S15** Details of cases in which diagnosis or suspicion of a specific major cardiac anomaly made in the first trimester was changed, in high‐risk populations
**Table S16** Screening characteristics of ultrasound in the first trimester for the detection of individual cardiac anomalies in non‐high‐risk populations
**Table S17** Screening characteristics of ultrasound in the first trimester for the detection of individual cardiac anomalies in high‐risk populations
**Table S18** Comparison of detection rates for individual cardiac anomalies in non‐high‐risk and high‐risk populations

## Data Availability

Data sharing is not applicable to this article as no new data were created or analyzed in this study.
