# Supplementary material for: First‐trimester ultrasound detection of fetal heart anomalies: systematic review and meta‐analysis
Source: Ultrasound Obstet Gynecol. 2022 Jan 5;59(1):11–25. doi: 10.1002/uog.23740 (PMC9305869; doi:10.1002/uog.23740)
Supplement: Supplementary file 1 — Appendix S1 Search strategy Appendix S2 QUADAS‐2 tool Appendix S3 Members of the Assessing Clinical and Cost Effectiveness of Prenatal first‐Trimester anomaly Screening (ACCEPTS) study group [file UOG-59-11-s001.docx]

**Appendix 1** Search Strategy

# The global search strategy involved two independent searches (A and B) combined with an “all” function. The search was conducted using MEDLINE, EMBASE, Web of Science and the Cochrane Library from January 1^st^, 1998 until July 17^th^, 2020.

**Medline:**

| **Search #** | **Searches conducted** |
| --- | --- |
| 1 | Ultrasonography, Prenatal/ |
| 2 | Prenatal diagnosis/ and exp ultrasonography/ |
| 3 | (ultrasound* or ultra-sound or ultrasonogra* or ultra-sonogra* or sonogra* or echocardiogra*).ti,ab. |
| 4 | ((fetal or foetal or fetus or foetus or prenat* or pre-nat* or prepart* or pre-part*) adj3 (screen* or scan* or structural assessment* or structural survey*)).ti,ab. |
| 5 | 1 or 2 or 3 or 4 |
| 6 | Pregnancy Trimester, First/ |
| 7 | (1st trimester or first trimester).ti,ab. |
| 8 | (early pregnan* or early gestation*).ti,ab. |
| 9 | ((10 week? or 11 week? or 12 week? or 13 week? or 14 week?) and (pregnan* or gestation* or fetal or foetal or fetus or foetus or prenat* or pre-nat* or prepart* or pre-part*)).ti,ab. |
| 10 | ((10week? or 11week? or 12week? or 13week? or 14week?) and (pregnan* or gestation* or fetal or foetal or fetus or foetus or prenat* or pre-nat* or prepart* or pre-part*)).ti,ab. |
| 11 | (((ten*2 or eleven*2 or twel*3 or thirteen*2 or fourteen*2) adj week?) and (pregnan* or gestation* or fetal or foetal or fetus or foetus or prenat* or pre-nat* or prepart* or pre-part*)).ti,ab. |
| 12 | 6 or 7 or 8 or 9 or 10 or 11 |
| 13 | exp *Congenital Abnormalities/ |
| 14 | (congenital* adj2 (defect? or malformation? or abnormalit* or anomal*)).ti,ab. |
| 15 | ((fetal or foetal or fetus or foetus) adj2 (defect? or malformation? or abnormalit* or anomal*)).ti,ab. |
| 16 | (structural adj2 (defect? or malformation? or abnormalit* or anomal*)).ti,ab. |
| 17 | ((non-chromosomal or nonchromosomal or chromosomal) adj2 (defect? or malformation? or abnormalit* or anomal*)).ti,ab. |
| 18 | neural tube defects/ or anencephaly/ or encephalocele/ or exp Spinal Dysraphism/ |
| 19 | craniofacial abnormalities/ or holoprosencephaly/ or cleft palate/ |
| 20 | Hernia, Umbilical/ |
| 21 | Gastroschisis/ |
| 22 | Bone Diseases, Developmental/ or Leg Length Inequality/ or limb deformities, congenital/ or exp polydactyly/ |
| 23 | exp "Transposition of Great Vessels"/ or Hypoplastic Left Heart Syndrome/ |
| 24 | exp heart septal defects/ or "tetralogy of fallot"/ |
| 25 | hernia, diaphragmatic/ or hernias, diaphragmatic, congenital/ |
| 26 | (acrania? or anencephaly or exencephaly or holoproscencephaly).ti,ab. |
| 27 | (encephalocele or ((brain or cereb*) adj bifid*)).ti,ab. |
| 28 | (omphalocele or exomphalos or (umbilical adj2 hernia?)).ti,ab. |
| 29 | gastroschisis.ti,ab. |
| 30 | megacystis.ti,ab. |
| 31 | (skelet* adj2 dysplasia?).ti,ab. |
| 32 | ((limb? or leg? or arm?) adj2 (short* or reduc* or inequality or unequal*)).ti,ab. |
| 33 | polydactyly.ti,ab. |
| 34 | (transpos* adj3 (great arteries or great vessel?)).ti,ab. |
| 35 | ((ventric* or heart) adj2 hypoplas*).ti,ab. |
| 36 | "tetralogy of fallot".ti,ab. |
| 37 | ((atrioventric* or atrio-ventric* or septal) adj2 defect?).ti,ab. |
| 38 | double outlet right ventric*.ti,ab. |
| 39 | spina bifida.ti,ab. |
| 40 | ((face or facial or lip* or palate*) adj2 cleft?).ti,ab. |
| 41 | (diaphragm* adj2 hernia*).ti,ab. |
| 42 | (((kidney or renal) adj2 agenesis) or potter* syndrome).ti,ab. |
| 43 | body stalk anomal*.ti,ab. |
| 44 | (club foot or club feet or talipes).ti,ab. |
| 45 | ventriculomegaly.ti,ab. |
| 46 | cystic hygroma?.ti,ab. |
| 47 | 13 or 14 or 15 or 16 or 17 or 18 or 19 or 20 or 21 or 22 or 23 or 24 or 25 or 26 or 27 or 28 or 29 or 30 or 31 or 32 or 33 or 34 or 35 or 36 or 37 or 38 or 39 or 40 or 41 or 42 or 43 or 44 or 45 or 46 |
| 48 | 5 and 12 and 47 |
| 49 | ((fetal or foetal or fetus or foetus) adj (anatomy or defect? or malformation? or abnormalit* or anomal*) adj5 (ultrasound* or ultra-sound or ultrasonogra* or ultra-sonogra* or sonogra* or echocardiogra* or scan* or screen* or survey* or assessment?)).ti,ab. |
| 50 | 12 and 49 |
| 51 | ((early pregnan* or early gestation* or 1st trimester or first trimester) adj3 (ultrasound* or ultra-sound or ultrasonogra* or ultra-sonogra* or sonogra* or echocardiogra* or scan* or screen* or survey* or assessment?)).ti,ab. |
| 52 | (((10 week? or 11 week? or 12 week? or 13 week? or 14 week?) adj3 (ultrasound* or ultra-sound or ultrasonogra* or ultra-sonogra* or sonogra* or echocardiogra* or scan* or screen* or survey* or assessment?)) and (pregnan* or gestation* or fetal or foetal or fetus or foetus or prenat* or pre-nat* or prepart* or pre-part*)).ti,ab. |
| 53 | (((10week? or 11week? or 12week? or 13week? or 14week?) adj3 (ultrasound* or ultra-sound or ultrasonogra* or ultra-sonogra* or sonogra* or echocardiogra* or scan* or screen* or survey* or assessment?)) and (pregnan* or gestation* or fetal or foetal or fetus or foetus or prenat* or pre-nat* or prepart* or pre-part*)).ti,ab. |
| 54 | (((ten*2 or eleven*2 or twel*3 or thirteen*2 or fourteen*2) adj week? adj3 (ultrasound* or ultra-sound or ultrasonogra* or ultra-sonogra* or sonogra* or echocardiogra* or scan* or screen* or survey* or assessment?)) and (pregnan* or gestation* or fetal or foetal or fetus or foetus or prenat* or pre-nat* or prepart* or pre-part*)).ti,ab. |
| 55 | 51 or 52 or 53 or 54 |
| 56 | 47 and 55 |
| 57 | 48 or 50 or 56 |
| 58 | exp animals/ not humans.sh. |
| 59 | 57 not 58 |

Embase

| [# ▲](http://ezproxy-prd.bodleian.ox.ac.uk:2843/sp-4.07.0b/ovidweb.cgi?&S=GJEOFPNJOJEBOOGOIPAKKGEHPEKHAA00&Sort+Sets=descending) | **Searches** |
| --- | --- |
| 1 | fetus echography/ |
| 2 | prenatal diagnosis/ and (echography/ or transvaginal echography/) |
| 3 | (ultrasound* or ultra-sound or ultrasonogra* or ultra-sonogra* or sonogra* or echocardiogra*).ti,ab. |
| 4 | ((fetal or foetal or fetus or foetus or prenat* or pre-nat* or prepart* or pre-part*) adj3 (screen* or scan* or structural assessment* or structural survey*)).ti,ab. |
| 5 | 1 or 2 or 3 or 4 |
| 6 | first trimester pregnancy/ |
| 7 | (1st trimester or first trimester).ti,ab. |
| 8 | (early pregnan* or early gestation*).ti,ab. |
| 9 | ((10 week? or 11 week? or 12 week? or 13 week? or 14 week?) and (pregnan* or gestation* or fetal or foetal or fetus or foetus or prenat* or pre-nat* or prepart* or pre-part*)).ti,ab. |
| 10 | ((10week? or 11week? or 12week? or 13week? or 14week?) and (pregnan* or gestation* or fetal or foetal or fetus or foetus or prenat* or pre-nat* or prepart* or pre-part*)).ti,ab. |
| 11 | (((ten*2 or eleven*2 or twel*3 or thirteen*2 or fourteen*2) adj week?) and (pregnan* or gestation* or fetal or foetal or fetus or foetus or prenat* or pre-nat* or prepart* or pre-part*)).ti,ab. |
| 12 | 6 or 7 or 8 or 9 or 10 or 11 |
| 13 | exp *congenital malformation/ |
| 14 | (congenital* adj2 (defect? or malformation? or abnormalit* or anomal*)).ti,ab. |
| 15 | ((fetal or foetal or fetus or foetus) adj2 (defect? or malformation? or abnormalit* or anomal*)).ti,ab. |
| 16 | (structural adj2 (defect? or malformation? or abnormalit* or anomal*)).ti,ab. |
| 17 | ((non-chromosomal or nonchromosomal or chromosomal) adj2 (defect? or malformation? or abnormalit* or anomal*)).ti,ab. |
| 18 | neural tube defect/ or anencephalus/ or encephalocele/ or exp spinal dysraphism/ or holoprosencephaly/ |
| 19 | cleft palate/ or cleft face/ or cleft lip/ or cleft lip palate/ |
| 20 | umbilical hernia/ |
| 21 | Gastroschisis/ |
| 22 | bone dysplasia/ or leg length inequality/ or polydactyly/ |
| 23 | great vessels transposition/ or hypoplastic left heart syndrome/ or exp heart septum defect/ |
| 24 | diaphragm hernia/ |
| 25 | kidney agenesis/ |
| 26 | (acrania? or anencephaly or exencephaly or holoproscencephaly).ti,ab. |
| 27 | (encephalocele or ((brain or cereb*) adj bifid*)).ti,ab. |
| 28 | (omphalocele or exomphalos or (umbilical adj2 hernia?)).ti,ab. |
| 29 | gastroschisis.ti,ab. |
| 30 | megacystis.ti,ab. |
| 31 | (skelet* adj2 dysplasia?).ti,ab. |
| 32 | ((limb? or leg? or arm?) adj2 (short* or reduc* or inequality or unequal*)).ti,ab. |
| 33 | polydactyly.ti,ab. |
| 34 | (transpos* adj3 (great arteries or great vessel?)).ti,ab. |
| 35 | ((ventric* or heart) adj2 hypoplas*).ti,ab. |
| 36 | "tetralogy of fallot".ti,ab. |
| 37 | ((atrioventric* or atrio-ventric* or septal) adj2 defect?).ti,ab. |
| 38 | double outlet right ventric*.ti,ab. |
| 39 | spina bifida.ti,ab. |
| 40 | ((face or facial or lip* or palate*) adj2 cleft?).ti,ab. |
| 41 | (diaphragm* adj2 hernia*).ti,ab. |
| 42 | (((kidney or renal) adj2 agenesis) or potter* syndrome).ti,ab. |
| 43 | body stalk anomal*.ti,ab. |
| 44 | (club foot or club feet or talipes).ti,ab. |
| 45 | ventriculomegaly.ti,ab. |
| 46 | cystic hygroma?.ti,ab. |
| 47 | 13 or 14 or 15 or 16 or 17 or 18 or 19 or 20 or 21 or 22 or 23 or 24 or 25 or 26 or 27 or 28 or 29 or 30 or 31 or 32 or 33 or 34 or 35 or 36 or 37 or 38 or 39 or 40 or 41 or 42 or 43 or 44 or 45 or 46 |
| 48 | 5 and 12 and 47 |
| 49 | ((fetal or foetal or fetus or foetus) adj (anatomy or defect? or malformation? or abnormalit* or anomal*) adj5 (ultrasound* or ultra-sound or ultrasonogra* or ultra-sonogra* or sonogra* or echocardiogra* or scan* or screen* or survey* or assessment?)).ti,ab. |
| 50 | 12 and 49 |
| 51 | ((early pregnan* or early gestation* or 1st trimester or first trimester) adj3 (ultrasound* or ultra-sound or ultrasonogra* or ultra-sonogra* or sonogra* or echocardiogra* or scan* or screen* or survey* or assessment?)).ti,ab. |
| 52 | (((10 week? or 11 week? or 12 week? or 13 week? or 14 week?) adj3 (ultrasound* or ultra-sound or ultrasonogra* or ultra-sonogra* or sonogra* or echocardiogra* or scan* or screen* or survey* or assessment?)) and (pregnan* or gestation* or fetal or foetal or fetus or foetus or prenat* or pre-nat* or prepart* or pre-part*)).ti,ab. |
| 53 | (((10week? or 11week? or 12week? or 13week? or 14week?) adj3 (ultrasound* or ultra-sound or ultrasonogra* or ultra-sonogra* or sonogra* or echocardiogra* or scan* or screen* or survey* or assessment?)) and (pregnan* or gestation* or fetal or foetal or fetus or foetus or prenat* or pre-nat* or prepart* or pre-part*)).ti,ab. |
| 54 | (((ten*2 or eleven*2 or twel*3 or thirteen*2 or fourteen*2) adj week? adj3 (ultrasound* or ultra-sound or ultrasonogra* or ultra-sonogra* or sonogra* or echocardiogra* or scan* or screen* or survey* or assessment?)) and (pregnan* or gestation* or fetal or foetal or fetus or foetus or prenat* or pre-nat* or prepart* or pre-part*)).ti,ab. |
| 55 | 51 or 52 or 53 or 54 |
| 56 | 47 and 55 |
| 57 | 48 or 50 or 56 |

Cochrane Library

| ID | Search |
| --- | --- |
| #1 | MeSH descriptor: [Ultrasonography, Prenatal] this term only |
| #2 | ultrasound* or ultra-sound or ultrasonogra* or ultra-sonogra* or sonogra* or echocardiogra*:ti,ab,kw (Word variations have been searched) |
| #3 | ((fetal or foetal or fetus or foetus or prenat* or pre-nat* or prepart* or pre-part*) near/3 (screen* or scan* or structural assessment* or structural survey*)):ti,ab,kw (Word variations have been searched) |
| #4 | #1 or #2 or #3 |
| #5 | MeSH descriptor: [Pregnancy Trimester, First] explode all trees |
| #6 | 1st trimester or "first trimester":ti,ab,kw (Word variations have been searched) |
| #7 | early pregnan* or "early gestation*":ti,ab,kw (Word variations have been searched) |
| #8 | ((("10 week*" or "11 week*" or "12 week*" or "13 week*" or "14 week*") and (pregnan* or gestation* or fetal or foetal or fetus or foetus or prenat* or pre-nat* or prepart* or pre-part*))):ti,ab,kw (Word variations have been searched) |
| #9 | (((10week* or 11week* or 12week* or 13week* or 14week*) and (pregnan* or gestation* or fetal or foetal or fetus or foetus or prenat* or pre-nat* or prepart* or pre-part*))):ti,ab,kw (Word variations have been searched) |
| #10 | ten week? or "eleven week?" or "twelve week?" or "thirteen week?" or "fourteen week?":ti,ab,kw (Word variations have been searched) |
| #11 | #5 or #6 or #7 or #8 or #9 or #10 |
| #12 | MeSH descriptor: [Congenital Abnormalities] explode all trees |
| #13 | ((congenital* near/2 (defect* or malformation* or abnormalit* or anomal*))):ti,ab,kw (Word variations have been searched) |
| #14 | (((fetal or foetal or fetus or foetus) near/2 (defect* or malformation* or abnormalit* or anomal*))):ti,ab,kw (Word variations have been searched) |
| #15 | ((structural near/2 (defect* or malformation* or abnormalit* or anomal*))):ti,ab,kw (Word variations have been searched) |
| #16 | (((non-chromosomal or nonchromosomal) near/2 (defect* or malformation* or abnormalit* or anomal*))):ti,ab,kw (Word variations have been searched) |
| #17 | (Acrania* or anencephaly or exencephaly or holoproscencephaly OR encephalocele or ((brain or cereb*) NEXT bifid*) OR omphalocele or exomphalos or (umbilical NEAR/2 hernia*) OR gastroschisis OR megacystitis OR (skelet* NEAR/2 dysplasia*) OR ((limb* or leg* or arm*) NEAR/2 (short* or reduc* or inequality or unequal*)) OR polydactyly OR (transpos* NEAR/3 ("great arteries" or "great vessel*")) OR ((ventric* or heart) NEAR/2 hypoplas*) OR "spina bifida" OR ((face or facial or lip* or palate*) NEAR/2 cleft*) OR (diaphragm* NEAR/2 hernia*) OR ((kidney or renal) NEAR/2 agenesis) or "potter* syndrome" OR "tetralogy of fallot " OR ((atrioventric* or atrio-ventric* or septal) near/2 defect*) OR "double outlet right ventric*" OR “Body Stalk Anomal*” OR “Club Foot” OR “Club Feet” OR Talipes OR Ventriculomegaly OR Cystic Hygroma*):ti,ab,kw |
| #18 | #12 or #13 or #14 or #15 or #17 |
| #19 | #4 and #11 and #18 |
| #20 | (((fetal or foetal or fetus or foetus) next (anatomy or defect* or malformation* or abnormalit* or anomal*) near (ultrasound* or ultra-sound or ultrasonogra* or ultra-sonogra* or sonogra* or echocardiogra* or scan* or screen* or survey* or assessment*))):ti,ab,kw (Word variations have been searched) |
| #21 | #11 and #20 |
| #22 | ((("early pregnan*" or "early gestation*" or 1st trimester or first trimester) near/3 (ultrasound* or ultra-sound or ultrasonogra* or ultra-sonogra* or sonogra* or echocardiogra* or scan* or screen* or survey* or assessment*))):ti,ab,kw (Word variations have been searched) |
| #23 | (((("10 week*" or "11 week*" or "12 week*" or "13 week*" or "14 week*") near/3 (ultrasound* or ultra-sound or ultrasonogra* or ultra-sonogra* or sonogra* or echocardiogra* or scan* or screen* or survey* or assessment*)) and (pregnan* or gestation* or fetal or foetal or fetus or foetus or prenat* or pre-nat* or prepart* or pre-part*))):ti,ab,kw (Word variations have been searched) |
| #24 | (((("ten week*" or "eleven week*" or "twelve week*" or "thirteen week*" or "fourteen week*") near/3 (ultrasound* or ultra-sound or ultrasonogra* or ultra-sonogra* or sonogra* or echocardiogra* or scan* or screen* or survey* or assessment*)) and (pregnan* or gestation* or fetal or foetal or fetus or foetus or prenat* or pre-nat* or prepart* or pre-part*))):ti,ab,kw (Word variations have been searched) |
| #25 | #22 or #23 |
| #26 | #18 AND #25 |
| #27 | #19 OR #21 OR #26 |

Web of Science Core Collection

| # 13 | #12 OR #8 |
| --- | --- |
| # 12 | #11 AND #4 |
| # 11 | #10 OR #9 |
| # 10 | TS=("fetal anatomy" OR "fetal defect*" OR "fetal malformation*" OR "fetal abnormalit*" OR "fetal anomal*" OR "foetal anatomy" OR "foetal defect*" OR "foetal malformation*" OR "foetal abnormalit*" OR "foetal anomal*") AND TS=(scan* OR survey* OR assessment? OR screen*) |
| # 9 | TS=("fetal anatomy" OR "fetal defect*" OR "fetal malformation*" OR "fetal abnormalit*" OR "fetal anomal*" OR "foetal anatomy" OR "foetal defect*" OR "foetal malformation*" OR "foetal abnormalit*" OR "foetal anomal*") AND TS=(ultrasound* or ultra-sound or ultrasonogra* or ultra-sonogra* or sonogra* or echocardiogra*) |
| # 8 | #7 AND #4 AND #1 |
| # 7 | #6 OR #5 |
| # 6 | TS=(acrania* or anencephaly or exencephaly or holoproscencephaly) OR TS=(encephalocele or ((brain or cereb*) NEXT bifid*)) OR TS=(omphalocele or exomphalos or (umbilical NEAR/2 hernia*)) OR TS=gastroschisis OR TS=megacystitis OR TS=(skelet* NEAR/2 dysplasia*) OR TS=((limb* or leg* or arm*) NEAR/2 (short* or reduc* or inequality or unequal*)) OR TS=polydactyly OR TS=(transpos* NEAR/3 ("great arteries" or "great vessel*")) OR TS=((ventric* or heart) NEAR/2 hypoplas*) OR TS="spina bifida" OR TS=((face or facial or lip* or palate*) NEAR/2 cleft*) OR TS=(diaphragm* NEAR/2 hernia*) OR TS=(((kidney or renal) NEAR/2 agenesis) or "potter* syndrome") OR TS=("tetralogy of fallot " OR ((atrioventric* or atrio-ventric* or septal) near/2 defect*) OR "double outlet right ventric*") OR TS= (“Body Stalk Anomal*” OR “Club Foot” OR “Club Feet” OR Talipes OR Ventriculomegaly OR Cystic Hygroma*) |
| # 5 | TS=(congenital* NEAR/2 (defect* or malformation* or abnormalit* or anomal*)) OR TS=(fetal NEAR/2 (defect* or malformation* or abnormalit* or anomal*)) OR TS= (foetal NEAR/2 (defect* or malformation* or abnormalit* or anomal*)) OR TS=(fetus NEAR/2 (defect* or malformation* or abnormalit* or anomal*)) OR TS=(foetus NEAR/2 (defect* or malformation* or abnormalit* or anomal*)) OR TS=(structural NEAR/2 (defect* or malformation* or abnormalit* or anomal*)) OR TS=(non-chromosomal NEAR/2 (defect* or malformation* or abnormalit* or anomal*)) OR TS=(nonchromosomal NEAR/2 (defect* or malformation* or abnormalit* or anomal*)) OR TS=(chromosomal NEAR/2 (defect* or malformation* or abnormalit* or anomal*)) |
| # 4 | #3 OR #2 |
| # 3 | TS=("1st trimester" or "first trimester") OR TS=("early pregnan*" or "early gestation*") OR TS=("10 week*" or "11 week*" or "12 week*" or "13 week*" or "14 week*") OR TS=(10week* or 11week* or 12week* or 13week* or 14week*) OR TS=("ten week*" OR "eleven week*" OR "twelve week*" OR "thirteen week*" OR "fourteen week*") |
| # 2 | TS=("1st trimester" or "first trimester") OR TS=("early pregnan*" or "early gestation*") |
| # 1 | TS=((pregnan* or gestation* or fetal or foetal or fetus or foetus or prenat* or pre-nat* or prepart* or pre-part*)) AND TS=(ultrasound* or ultra-sound or ultrasonogra* or ultra-sonogra* or sonogra* or echocardiogra*) |

# **Appendix 2** QUADAS-2 Assessment Tool

**Defining the review question:**

1. What is the sensitivity of first trimester ultrasound for the detection of cardiac malformations?

2. What factors might impact detection rates?

- Patient selection: pregnant women with gestational age prior to 14^+6^ weeks, mothers with all levels of risk and with either singleton or multiple pregnancies were included
- Index Test: Transvaginal and/or Transabdominal 2D Ultrasound prior to 14^+6^ weeks gestational age.
- Reference Standard: Postnatal examination of fetus or postmortem of fetus for evidence/confirmation of structural abnormalities.
- Target condition: congenital cardiac abnormalities.

**Domain 1: Patient Selection**

| 1. **Risk of Bias:** Could the selection of patients have introduced bias? | LOW/HIGH/UNCLEAR |
| --- | --- |
| Signaling Questions: |  |
| 1. Was a consecutive (vs. random sample) of patients enrolled? | YES/NO/UNCLEAR |
| ii. Did the study avoid inappropriate exclusions? | YES/NO/UNCLEAR |
| 1. **Applicability:** Are there concerns that the included patients and setting do not match the review question (i.e. severity of the target condition, demographic features, presence of co-morbidity, setting)? | LOW/HIGH/UNCLEAR |

**Domain 2: Index Test**

| 1. **Risk of Bias:** Could the conduct or interpretation of the index test have introduced bias? | LOW/HIGH/UNCLEAR |
| --- | --- |
| Signaling Questions: |  |
| 1. Were sonographers blinded to the history (risk profile) of the patients? | YES/NO/UNCLEAR |
| 1. Were all of the included first trimester scans performed prior to 14^+6^ weeks gestational age? | YES/NO/UNCLEAR |
| 1. **Applicability:** Are there concerns that the index test, its conduct, or interpretation differ from the review question? | LOW/HIGH/UNCLEAR |

**Domain 3: Reference Standard**

| 1. **Risk of Bias:** Could the reference standard, its conduct, or its interpretation have introduced bias? | LOW/HIGH/UNCLEAR |
| --- | --- |
| Signaling Questions: |  |
| i. Was an appropriate reference standard used to correctly classify the target condition? | YES/NO/UNCLEAR |
| 1. Were the reference standard results interpreted without knowledge of the results of the index test? | YES/NO/UNCLEAR |
| 1. **Applicability:** Are there concerns that the target condition as defined by the reference standard does not match the question? | LOW/HIGH/UNCLEAR |

**Domain 4: Flow and Timing**

| 1. **Risk of Bias:** Could the patient flow have introduced bias? | LOW/HIGH/UNCLEAR |
| --- | --- |
| Signaling Questions: |  |
| 1. Did all patients included in the study undergo examination with the reference standard? (either postnatal examination for live-births or post-mortem for still-births/TOPs in those with diagnosed malformations). | YES/NO/UNCLEAR |
| ii.Were all patients enrolled in the study included in the analysis? | YES/NO/UNCLEAR |
| iii.Were all measures of 1^st^ trimester ultrasound detection accuracy (eg. TP, FP, TN, FN) reported? | YES/NO/UNCLEAR |

# **Appendix 3** Members of the Assessing Clinical and Cost Effectiveness of Prenatal first-Trimester anomaly Screening (ACCEPTS) study group

**Clinical and study design group:**

Aris T Papageorghiou (PI, University of Oxford, UK), Zarko Alfirevic (Liverpool Women’s NHS Foundation Trust, UK), Trish Chudleigh (Cambridge University Hospitals NHS Foundation Trust, UK), Hilary Goodman (Hampshire Hospital NHS Foundation Trust, UK), Christos Ioannou (Oxford University Hospitals NHS Trust, UK), Heather Longworth (Liverpool Women’s NHS Foundation Trust, UK), Jehan N Karim (University of Oxford, UK), Kypros H Nicolaides (Fetal Medicine Research Institute, King’s College Hospital, UK), Pranav Pandya (University College London Hospitals NHS Foundation Trust, UK), Gordon Smith (University of Cambridge, UK), Basky Thilaganathan (St George's University Hospitals NHS Foundation Trust, UK) and Jim Thornton (University of Nottingham, UK).

**Health economics group:** Oliver Rivero-Arias (Lead, University of Oxford, UK), Helen Campbell (University of Oxford, UK), Ed Juszczak (University of Oxford, UK), Louise Linsell (University of Oxford, UK) and Ed Wilson (University of Cambridge, UK).

**Qualitative research:** Lisa Hinton (University of Cambridge, UK)

**Patient Voice:** Jane Fisher (Lead, Antenatal Results and Choices, UK), Elizabeth Duff (National Childbirth Trust, UK), Anne Rhodes (Tiny Tickers, UK), Gil Yaz (SHINE UK).
