# Supplementary material for: First‐trimester ultrasound detection of fetal heart anomalies: systematic review and meta‐analysis
Source: Ultrasound Obstet Gynecol. 2022 Jan 5;59(1):11–25. doi: 10.1002/uog.23740 (PMC9305869; doi:10.1002/uog.23740)
Supplement: Supplementary file 2 — Table S1 Characteristics of studies reporting on the detection of major cardiac anomalies by first‐trimester ultrasound in non‐high‐risk populations Table S2 Characteristics of studies reporting on the detection of major cardiac anomalies by first‐trimester ultrasound in high‐risk populations Table S3 Details of anatomical protocols used by studies evaluating non‐high‐risk populations Table S4 Details of anatomical protocols used by studies evaluating high‐risk populations Table S5 Number of major cardiac anomalies diagnosed or suspected in the first trimester with independent secondary confirmation, in non‐high‐risk populations Table S6 Number of major cardiac anomalies diagnosed or suspected in the first trimester with independent secondary confirmation, in high‐risk populations Table S7 Impact of first‐trimester imaging protocol on the detection of major cardiac anomalies in high‐risk populations Table S8 Characteristics of major cardiac anomalies diagnosed following first‐trimester ultrasound assessment in non‐high‐risk populations Table S9 Characteristics of major cardiac anomalies suspected following first‐trimester ultrasound assessment in non‐high‐risk populations Table S10 Characteristics of major cardiac anomalies reported as cardiac abnormality of unknown significance (AUS) following first‐trimester ultrasound assessment in non‐high‐risk populations Table S11 Details of cases in which diagnosis or suspicion of a specific major cardiac anomaly made in the first trimester was changed, in non‐high‐risk populations Table S12 Characteristics of major cardiac anomalies diagnosed following first‐trimester ultrasound assessment in high‐risk populations Table S13 Characteristics of major cardiac anomalies suspected following first‐trimester ultrasound assessment in high‐risk populations Table S14 Characteristics of major cardiac anomalies reported as cardiac abnormality of unknown significance (AUS) following first‐trimester ultrasound assessment in high‐risk popu [file UOG-59-11-s002.docx]

**Table S1** Characteristics of studies reporting on the detection of major cardiac anomalies by first-trimester ultrasound in non-high-risk populations

| **Study**  **(year)** | **Fetuses (n)** | **Prevalence of Major Cardiac Anomalies**  **(n per 100 fetuses)** | **GA (weeks) or CRL (mm)** | **Population & Recruitment Characteristics** | **Healthcare Setting** | **Aneuploid Fetuses Included?**  **(%)*** | **Index Test**** | **Sonographer Experience** |
| --- | --- | --- | --- | --- | --- | --- | --- | --- |
| Whitlow 1999^31^ | 6634 | 0.18  (0.09 – 0.32) | 11-14^+6^ | Unselected, consecutive recruitment | University Hospital | Yes (0.7%) | TA/TV (20.1)^ | 6 clinicians & 4 sonographers. All trained in first trimester US |
| Michailidis 2001^32^ | 6650 | 0.15  (0.07 – 0.28) | 38-84 | Unselected, consecutive recruitment, prospective study | University Hospital | No | TA/TV (14%)^ | NA |
| McAuliffe 2005^33^ | 325 | 0.62  (0.07 – 2.21) | 11-13^+6^ | Unselected, singleton pregnancies only, prospective study | University Hospital, Tertiary Care | No | TA/TV  (24.6%)^ | NA |
| Cedergren 2006^34^ | 2708 | 0.11  (0.02 – 0.32) | 11-14 | Unselected, consecutive recruitment | University Hospital | Yes (0.3%) | TA | Midwife sonographers with at least ten years experience |
| Souka 2006^35^ | 1148 | 2.54  (1.92 – 3.29) | 11-14 | Unselected | Unclear | Yes | TA/TV^ | NA |
| Srisupundit 2006^36^ | 597 | 0.34  (0.04 – 1.20) | 11-14 | Unselected women attending NT scan, singleton pregnancies only, prospective study | University Hospital | Yes | TA | NA |
| Vimpelli 2006^37^ | 584 | 1.03  (0.38 – 2.22) | 11-13^+6^ | Unselected | Unclear | Yes | TV | NA |
| Dane 2007^38^ | 1290 | 0.31  (0.08 – 0.79) | 11-14 | Unselected | Research Hospital | Yes | TA/TV^ | 2 operators with 6 and 2 years experience respectively |
| Lombardi 2007^39^ | 623 | 0.48  (0.10 – 1.40) | 12^+3^-13^+6^ | Unselected women attending routine NT scan; singleton pregnancies only | Unclear | Yes | TA | NA |
| Chen 2008^40^  (control group) | 3693 | 0.48  (0.10 – 0.77) | 10-14^+6^ | Unselected, singleton pregnancies only, consecutively randomized (RCT) | One university & one regional hospital | Yes | TA/TV^ | 8 experienced operators |
| Chen 2008^40^  (study group) | 3949 | 0.43  (0.25 – 0.69) | 12-14^+6^ | Unselected, singleton pregnancies only, consecutively randomized (RCT) | One university & one regional hospital | Yes | TA/TV^ | 8 experienced operators |
| Li 2008^41^ | 2232 | 0.22  (0.07 – 0.52) | 11-14 | Unselected, consecutive recruitment | Unclear | Yes | TA/TV^  (2.0%) | NA |
| Bennasar 2009^42^ | 64 | 17.19  (8.90 – 28.68) | 11-14^+6^ | Mixed cohort (majority unselected combined with high risk women), singleton pregnancies only, prospective study | University Hospital | Yes | TV | ‘Non-expert’ operators trained in first trimester US and fetal echocardiography |
| Oztekin 2009^43^ | 1085 | 0.28  (0.06 – 0.81) | 11-14 | Unselected | Research Hospital | Yes | TA/TV^ | Single sonographer |
| Abu Rustum 2010^44^ | 1370 | 0.80  (0.40 – 1.43) | 11-13^+6^ | Unselected, retrospective study | Unclear | Yes | TA/TV^ | Single sonographer with FMF certification |
| Sinkovskaya 2010^45^ | 100 | 8.00  (3.52 – 15.16) | 11-14^+6^ | Consecutive recruitment; singleton pregnancies only; prospective study | Unclear | Yes | TA/TV^ (19%) | NA |
| Hartge 2011^46^ | 3521 | 2.87  (2.34 – 3.47) | 11-13^+6^ | Mixed high risk and low risk population, singleton pregnancies only, retrospective study | Tertiary referral centre; | Yes | TA/TV^ (35.8%) | NA |
| Jakobsen 2011^47^ | 9324 | 0.46  (0.33 – 0.62) | 11-14 | Unselected, retrospective study | University Hospital | Yes | TA/TV^ | NA |
| Krapp 2011^48^ | 690 | 2.75  (1.67 – 4.27) | 45-84 | Mixed high and low risk population, retrospective study | Unclear | Yes | TA/TV^ (5.2%) | NA |
| Syngelaki 2011^17^ | 44,859 | 0.26  (0.21 – 0.31) | 11-13 | Unselected, singleton pregnancies only (presumed euploid), retrospective study, | Multicentre (3) including tertiary care referral centre | No | TA/TV^  (1%) | NA |
| Volpe 2011^49^ | 4445 | 0.58  (0.38 – 0.86) | 45-84 | Unselected, prospective cohort | Single centre, University Hospital | Yes | TA/TV^ (7.3%) | Sonographers with extensive experience, FMF certified. |
| Becker 2012^50^ | 6544 | 0.23  (0.13 – 0.38) | 11-13^+6^ | Women with normal NT only (≤ 95th centile), prospective, consecutive recruitment | University Hospital | Yes (0.6%)^$^ | TA/TV^ (23.4%) | Single examiner with 10 years experience |
| Eleftheriades 2012^51^ | 3774 | 0.90  (0.62 – 1.26) | 11-13^+6^ | Unselected fetuses undergoing routine prospective ultrasound | Private Fetal Medicine Unit | Yes | TA | Obstetrician with extensive experience & FMF certificate. In case of abnormality, further examination by fetal cardiologist. |
| Grande 2012^52^ | 13,723 | 0.27  (0.19 – 0.37) | 11-14 | Mixed (majority low risk scans, 13% for raised NT), singleton pregnancies only, retrospective study | Tertiary Care Centre | No | TA/TV | 19 Obstetricians |
| Novotna 2012^53^ | 9150 | 0.20  (0.12 – 0.31) | 11-14 | Unselected, prospective study | Single centre | Yes | TA/TV | 23 operators with minimun 2 years experience. |
| Pilalis 2012^54^ | 3902 | 0.28  (0.14 – 0.50) | 11-14 | Unselected, retrospective study | Private maternity hospital | Yes | TA/TV^ | FMF certified; 2 years special training in ultrasound. |
| Iliescu 2013^55^ | 5472 | 0.62  (0.43 – 0.87) | 12-13^+6^ | Unselected, prospective study | University Hospital | Yes (0.4%) | TA/TV^ (7.8%) | Obstetricians specializing in prenatal diagnosis with at least 5 years accreditations and specific training for early fetal cardiac assessment. |
| Wang 2013^56^ | 2822 | 0.35  (0.17 – 0.65) | 11-14 | Not stated | University Hospital | Yes | TA | 5 Experienced obstetric sonographers |
| Orlandi 2014^57^ | 4820 | 0.44  (0.27 – 0.67) | 11-14 | Unselected, singleton pregnancies only, prospective study | Centre for prenatal diagnosis | Yes | TA/TV^ (5%) | 3 Experienced sonographers with FMF certificates for NT, NB, TR, DV. |
| Andrew2015^58^ | 4421 | 0.07  (0.01 – 0.20) | 11-14 | Unselected, consecutive recruitment, retrospective study | Tertiary referral centre; | Yes | TA/TV^ | 4 operators with NT certification |
| Colosi 2015^59^ | 5924 | 0.05  (0.01 – 0.15) | 11-13^+6^ | Unselected, singleton pregnancies only, prospective study | Fetal Medicine Unit | Yes (4.7%) | TA/TV (1.9%)^ | 4 operators with FMF Certification |
| Wiechec 2015^60^ | 1084 | 3.41  (2.41 – 4.67) | 11-13^+6^ | Unselected, prospective study | University Hospital Clinic | Yes (6.6%) | TA/TV  (5.25%)^ | NA |
| Takita 2016^61^ | 2028 | 0.74  (0.41 – 1.22) | 11-13^+6^ | Unselected, singleton pregnancies only, prospective study | University Hospital | Yes (0.6%) | TA | NA |
| Tudorache 2016^62^ | 3240 | 0.99  (0.68 – 1.39) | 11^+2^-13^+4^ | Unselected, prospective, consecutive recruitment | University Hospital, Tertiary referral centre | Yes | TA | NA |
| De Robertis 2017^63^ | 5343 | 0.62  (0.43 – 0.87) | 45-84 | Unselected, singleton pregnancies only, consecutive recruitment, prospective study. Excluded all pregnancies which underwent TOP for cardiac anomaly in the first trimester. | Tertiary Care | Yes | TA/TV (7%) | Expert sonographers, FMF certified |
| Vellamkondu 2017^64^ | 440 | 0.91  (0.25 – 2.31) | 11-14 | Unselected, singleton pregnancies only, prospective study | University Hospital, Tertiary care | Yes (0.5%) | TA/TV | NA |
| Fernandez 2018^65^ | 663 | 0.75  (0.25 – 1.75) | 11-13^+6^ | Low risk singleton pregnancies only, prospective study | Fetal Medicine Unit | Yes | TV/TA | 2 sonographers with >10 years experience |
| Kenkhuis 2018^66^ | 5534 | 0.23  (0.13 – 0.40) | 11-13^+6^ | Unselected women offered Combined Test for Aneuploidy screening (n=5237) and women at a priori high risk of fetal anomalies (297) | 2 Referral centres; 6 community ultrasound practices | Yes | TA/TV^ | Sonographers given specific first trimester US training |
| Sainz 2018^67^ | 504 | 2.98  (1.68 – 4.87) | 11-14^+6^ | Mixed low risk (n = 433) and high-risk population (n = 71), singleton pregnancies only, prospective study | University Hospital | Yes | TA | 2 sonographers: one with >5 years obstetric US experience, one with SESEGO Level 3 training but <1 year experience. |
| Vayna 2018^68^ | 6114 | 0.51  (0.34 – 0.72) | 11-14 | Unselected, retrospective study | University Hospital | Yes | TA/TV^ | NA |
| Zheng 2018^69^ | 1592 | 1.88  (1.27 – 2.68) | 45 - 84 | Unselected women presenting for NT scan, consecutive recruitment | University Hospital | Yes | TA/TV^ | 2 Sonographers with FMF certification |
| Chen 2019^70^ | 10,294 | 1.18  (0.98 – 1.40) | 11-13^+6^ | Low risk cohort, prospective study, | Single centre | Yes | NA | Sonographers with DEGUM II Certificate |
| Duta 2019^71^ | 7693 | 0.44  (0.31 – 0.62) | 11-14 | Unselected, retrospective study of prospectively, consecutively collected data | Fetal Medicine Unit, Single centre | No | TA/TV^ | 8 sonographers certified for 11-14 week scan |
| Ebrashy 2019^72^ | 3400 | 2.94  (2.40 – 3.57) | 11-13^+6^ | Unselected, prospective study | Fetal Medicine Unit, University Hospital | Yes | TA/TV^ (31.3%) | Fetal medicine specialists with FMF Certification |
| Erenel 2019^73^ | 707 | 1.70  (0.88 – 2.95) | 11-14 | Prospective, Unselected | Perinatology clinic affiliated with University and Research Hospital | Yes | TA/TV^ (4.6%) | 5 clinicians with experience in first trimester ultrasound |
| Syngelaki 2019^74^ | 101,793 | 0.35  (0.31 – 0.39) | 11-13^+6^ | Unselected, singleton pregnancies only (presumed euploid), retrospective study of prospectively collected data, | 2 University Hospitals (One Tertiary care, one regional) | No | TA/TV^  (3%) | 476 Sonographers with FMF Certification |

Only first author given for each study. Total number of fetuses included in this subgroup n = 306,872. Pooled prevalence of major cardiac anomalies (n per 100 fetuses) in this subgroup was 0.41% (fixed-effects model, 95% CI 0.39 – 0.43%). *In studies where aneuploid fetuses were included, percentage of the study population confirmed as aneuploid by karyotyping has been indicated in parentheses. **In studies where both transabdominal (TA) and transvaginal (TV) ultrasound were used, the number in parentheses refers to the percentage of the study population who received screening with both screening tests. ^Studies where TV ultrasound was performed only in situations when visualization with TA was suboptimal. ^$^Only known euploid fetuses included in this meta-analysis as insufficient data provided on entire study cohort. NB – Nasal bone examination. NT – Nuchal translucency examination. DEGUM –German Society of Ultrasound in Medicine and Biology. DV – Ductus Venosus examination. FMF – Fetal Medicine Foundation. SESEGO – Spanish Society of Gynecology and Obstetrics (SEGO) ultrasonography certification. TR – Tricuspid regurgitation examination. US – Ultrasound.

**Table S2** Characteristics of studies reporting on the detection of major cardiac anomalies by first-trimester ultrasound in high-risk populations

| **Study**  **Year** | **Fetuses (n)** | **Prevalence of Major Cardiac Anomalies**  **(n per 100 fetuses)** | **GA (weeks) or CRL (mm)** | **Population & Recruitment Characteristics** | **Healthcare Setting** | **Aneuploid Fetuses Included?**  **(%)*** | **Index Test**** | **Sonographer Experience** |
| --- | --- | --- | --- | --- | --- | --- | --- | --- |
| Carvalho 1998^16^ | 15 | 26.67  (7.79 – 55.10) | 11-13^+6^ | Women with history of cardiac anomaly and/or raised NT | Tertiary Care | Yes | TA | NA |
| Zosmer  1999^75^ | 112 | 8.93  (4.37 – 15.81) | 11-13^+6^ | Fetuses with NT ≥ 99% centile or ≥ 3.5mm, with normal CVS result | Tertiary Care | No | TA/TV (5%) | Expert in fetal echocardiography |
| Comas Gabriel 2002^76^ | 200 | 16.50  (11.64 – 22.38) | 11-14 | Women with increased a priori risk for cardiac anomalies | Multi-centre (3), FM Referral Centres | Yes | TV/TA | 3 experienced operators |
| Den Hollander 2002^77^ | 101 | 2.97  (0.62 – 8.44) | 11-14 | Women with previous infant affected with CHD (92%) and/or parental consanguinity | Tertiary Care | Yes | TA/TV^ | NA |
| Haak  2002^78^ | 45 | 35.56  (21.87 – 51.22) | 11-14 | Fetuses with NT>95%, singleton pregnancies only, consecutive recruitment, prospective study | Tertiary Care | Yes | TV | Single experienced examiner |
| Huggon 2002^79^ | 478 | 16.11  (12.93 – 19.72) | 40 - 84 | Women with increased NT (≥ 4mm) and/or abnormal DV Flow; Women with first degree relative with CHD; Suspicion of CHD or extra-cardiac anomaly at 10-14 weeks scan | Tertiary Referral Centre | Yes | TA/TV^ (<1%) | 2 experienced sonographers (one fetal cardiologist; one gynaecologist with specific experience in fetal echocardiography) |
| Weiner  2002^80^ | 392 | 1.79  (0.72 – 3.64) | 11-14 | High risk patients undergoing fetal echocardiography (predominantly for maternal diabetes and previous pregnancy affected by CHD) | Unclear | Yes | TV/TA^ | NA |
| Chen  2004^81^ | 1609 | 0.87  (0.47 – 1.45) | 11-14 | High risk women aged 35 or older | University Hospital | Yes | TA/TV^ | 2 operators with >10 years experience each |
| Bronshtein 2008^82^ | 23 | 34.78  (16.38 – 57.27) | 11-14 | Fetuses with increased NT  (≥ 3.5mm) | Unclear | Yes | TV | NA |
| Weiner  2008^83^ | 200 | 11.00  (7.02 – 16.18) | 11-13^+6^ | Fetuses with NT ≥ 3mm or cystic hygroma | Unclear | Yes | TV | Specialists in MFM trained in fetal echocardiography |
| Persico  2011^84^ | 886 | 6.32  (4.81 – 8.13) | 11-13^+6^ | Patients with US exam prior to CVS (majority for increased risk after Combined Screening), prospective study | Tertiary Care | Yes | TA | Obstetricians with extensive experience in T2 anomaly scanning and T1 US; Images reviewed by fetal cardiologist. |
| Volpe  2012^85^ | 870 | 6.55  (5.00 – 8.41) | 11-13^+6^ | Women at high risk for CHD. Fetuses who underwent ECHO at both 11-14 and 18-22 weeks. Retrospective study | FM Referral Centre | Yes | TA/TV^ (9%) | NA |
| Votino  2012^86^ | 15 | 13.33  (1.66 – 40.46) | 11^+2^-13^+4^ | High risk women: Maternal CHD, Maternal risk factors, maternal diabetes, NT>95%, Abnormal TR, DV. Singleton pregnancies only, prospective study | Single Centre; University Hospital; Fetal medicine unit. | Yes | TA | Single trained operator |
| Miller  2013^87^ | 341 | 2.40  (0.97 – 4.86) | 45-84 | Women with pre-gestational diabetes, prospective, observational study | University Hospital | Yes | TA/TV | Sonographers certified by FMF or NT Quality Review Program |
| Zidere  2013^88^ | 1200 | 6.08  (4.80 – 7.59) | 11-14 | Fetuses undergoing detailed fetal echocardiography for various indications: raised NT, family history, extracardiac malformation, co-twin affected by anomaly | Tertiary Care | Yes | TA/TV^ | Single specialist fetal cardiologist |
| D'Antonio 2016^89^ | 2128 | 0.56  (0.29 – 0.98 | 11-13^+6^ | Consecutive twin pregnancies, retrospective cohort analysis, | Tertiary Care | Yes | TA/TV^ | NA |
| Zalel  2017^90^ | 43 | 62.79  (46.73 – 77.02) | 11-13^+6^ | Fetuses with NT>99% centile (>3.4mm), prospective study | Tertiary care affiliated centre | Yes | TV | Single experienced examiner |
| Syngelaki  2020^91^ | 12,732 | 0.41  (0.31 – 0.54) | 11-14 | Twin pregnancies (MCDA and DCDA), retrospective cohort analysis | Multi-centre (3) | No | TA/TV^  (3%) | Sonographers certified by the Fetal Medicine Foundation |

Only first author given for each study. Total number of fetuses included in this subgroup n = 21,390. Pooled prevalence of major cardiac anomalies (n per 100 fetuses) in this subgroup was 1.36 (fixed-effects model, 95% CI 1.20 – 1.52%) **In studies where both transabdominal (TA) and transvaginal (TV) ultrasound were used, the number in parentheses refers to the percentage of the study population who received screening with both screening tests. ^Studies where second listed ultrasound modality was performed only in situations when visualization with the first modality was suboptimal. CHD – Congenital Heart Disease. CVS – Chorionic Villus Sampling. DV – Ductus Venosus examination. DCDA – Dichorionic Diamniotic Twin pregnancy. FMF – Fetal Medicine Foundation. MCDA – Monochorionic Diamniotic Twin pregnancy. NB – Nasal bone examination. NT – Nuchal translucency examination.TR – Tricuspid regurgitation examination. US – Ultrasound.

**Table S3** Details of anatomical protocols used by studies evaluating non-high-risk populations

| Study | No Protocol Used | Situs | Cardiac Axis | Four Chamber View | Outflow/Inflow Tract Assessment^ | Colour Flow Doppler | Pulsed Doppler | Ductus Venosus Assessment | Tricuspid Valve Assessment | Fetal ECHO* | Protocol Analysis Group** |
| --- | --- | --- | --- | --- | --- | --- | --- | --- | --- | --- | --- |
| Whitlow 1999^31^ | x | x | x | ✓ | x | x | x | x | x | x | 2 |
| Michailidis 2001^32^ | x | x | x | ✓ | x | x | x | x | x | x | 2 |
| McAuliffe 2005^33^ | x | ✓ | x | ✓ | x | x | x | x | x | x | 2 |
| Cedergren 2006^34^ | ✓ | x | x | x | x | x | x | x | x | x | 1 |
| Souka 2006^35^ | x | x | x | ✓ | 3VV (pulmonary artery, aorta and superior vena cava) | x | x | x | x | x | 4 |
| Srisupundit 2006^36^ | No details regarding protocol used in study provided by authors. | | | | | | | | | | |
| Vimpelli 2006^37^ | x | ✓ | x | ✓ | Longitudinal views of the aorta and pulmonary trunk, crossing of aorta and pulmonary trunk (and/or 3VV), aortic arch, ductal arch | x | x | x | x | ✓ | 4 |
| Dane 2007^38^ | ✓ | x | x | x | x | x | x | x | x | x | 1 |
| Lombardi 2007^39^ | x | ✓ | ✓ | ✓ | Crossing of the main pulmonary artery with the aorta; straight line of the pulmonary artery surrounded by aortic arch; connection of the aorta and ductus arteriosus | ✓ | x | x | ✓ | ✓ | 5 |
| Chen 2008^40^  (control group) | ✓ | x | x | x | x | x | x | x | x | x | 1 |
| Chen 2008^40^  (study group) | x | x | x | ✓ | Aortic and pulmonary outflow tracts | x | x | x | x | x | 4 |
| Li 2008^41^ | ✓ | x | x | x | x | x | x | x | x | x | 1 |
| Bennasar 2009^42^ | x | ✓ | x | ✓ | Continuity between the aortic root and the interventricular septum; pulmonary trunk in a short-axis view, pulmonary branching; Crossing-over of the aorta and pulmonary trunk. 3VVT. | ✓ | x | x | x | ✓ | 5 |
| Oztekin 2009^43^ | x | x | ✓ | ✓ | Examination of great vessels | x | x | x | x | x | 4 |
| Abu Rustum 2010^44^ | x | ✓ | x | ✓ | x | x | ✓ | x | ✓ | x | 2 |
| Sinkovskaya 2010^45^ | x | x | ✓ | ✓ | Imaging of the outflow tracts | x | x | x | x | x | 4 |
| Hartge 2011^46^ | x | ✓ | x | ✓ | Fetal echocardiography carried out using standardized anatomical transverse and longitudinal planes | ✓ | ✓ | ✓ | x | ✓ | 5 |
| Jakobsen 2011^47^ | ✓ | x | x | x | x | x | x | x | x | x | 1 |
| Krapp 2011^48^ | x | ✓ | x | ✓ | Pulmonary vein inflow into left atrium; outflow of the aorta from the left ventricle; 3VV (outflow of the main pulmonary artery from right ventricle); transverse aortic arch; branching of the brachiocephalic trunk, left common carotid and left subclavian artery. | ✓ | x | x | x | ✓ | 5 |
| Syngelaki 2011^17^ | x | x | x | ✓ | x | x | ✓ | x | ✓ | x | 2 |
| Volpe 2011^49^ | x | ✓ | x | ✓ | Left ventricular outflow tract; Right ventricular outflow tract; Crossover of the great arteries; 3VVT. | ✓ | x | x | x | x | 5 |
| Becker 2012^50^ | x | ✓ | x | ✓ | Visualization of inflow and outflow tracts | ✓ | x | x | x | ✓ | 5 |
| Eleftheriades 2012^51^ | x | ✓ | x | ✓ | x | x | x | x | x | x | 2 |
| Grande 2012^52^ | x | x | x | ✓ | x | x | ✓ | ✓ | x | x | 2 |
| Novotna 2012^53^ | ✓ | x | x | x | x | x | x | x | x | x | 1 |
| Pilalis 2012^54^ | ✓ | x | x | x | x | x | x | x | x | x | 1 |
| Iliescu 2013^55^ | x | ✓ | x | ✓ | Aorta arising from left ventricle and pulmonary trunk arising from right ventricle and crossing to fetal left side over ascending aorta; 3VVT; | ✓ | x | ✓ | x | x | 5 |
| Wang 2013^56^ | ✓ | x | x | x | x | x | x | x | x | x | 1 |
| Orlandi 2014^57^ | x | ✓ | x | ✓ | Origin of aorta from left ventricle; Origin of pulmonary artery from right ventricle, and vessels crossing | ✓ | x | x | x | x | 5 |
| Andrew2015^58^ | x | ✓ | x | ✓ | Examination of great vessels | ✓ | ✓ | ✓ | ✓ | x | 5 |
| Colosi 2015^59^ | x | x | x | ✓ | x | x | ✓ | ✓ | x | x | 2 |
| Wiechec 2015^60^ | x | x | x | ✓ | 3VVT: number of arterial arms, subjective assessment of their size ration and flow direction | ✓ | x | x | x | x | 5 |
| Takita 2016^61^ | x | ✓ | x | ✓ | x | x | x | x | x | x | 2 |
| Tudorache 2016^62^ | x | ✓ | x | ✓ | 3VVT; Outflow tract crossing | ✓ | x | ✓ | ✓ | x | 5 |
| De Robertis 2017^63^ | x | ✓ | x | ✓ | 3VVT | ✓ | x | x | x | x | 5 |
| Vellamkondu 2017^64^ | No details regarding protocol used in study provided by authors. | | | | | | | | | | |
| Fernandez 2018^65^ | x | ✓ | x | ✓ | Axial section at level of great vessels showing pulmonary artery, aorta and vena cava | x | x | x | x | x | 4 |
| Kenkhuis 2018^66^ | x | ✓ | x | ✓ | x | ✓ | ✓ | x | x | x | 3 |
| Sainz 2018^67^ | x | x | x | ✓ | Aortic and pulmonary outflow tracts | x | x | x | x | x | 4 |
| Vayna 2018^68^ | x | ✓ | x | ✓ | Origin of the aorta and pulmonary artery; 3VV; aortic arch; right subclavian artery | ✓ | x | ✓ | ✓ | x | 5 |
| Zheng 2018^69^ | x | x | ✓ | ✓ | 3VVT | ✓ | x | x | x | x | 5 |
| Chen 2019^70^ | x | x | x | ✓ | Evaluation of major vessels | x | x | x | x | x | 5 |
| Duta 2019^71^ | x | ✓ | ✓ | ✓ | Aorta, pulmonary artery, 3VVT, and subclavian artery | ✓ | x | x | x | x | 5 |
| Ebrashy 2019^72^ | x | x | ✓ | ✓ | Examination of great arteries, (vessel diameter, crossing); aortic arch; ductal arch | ✓ | x | x | x | ✓ | 5 |
| Erenel 2019^73^ | x | ✓ | x | ✓ | 3VV | ✓ | x | x | x | x | 5 |
| Syngelaki 2019^74^ | x | x | x | ✓ | Examination of outflow tracts | ✓ | ✓ | x | ✓ | x | 5 |

(✓) Identifies anatomical views and/or examinations included in the study protocol.

(x) Identifies anatomical views and/or examinations which were not routinely reported as being included in the study protocol.

*Identifies studies which have described their first trimester cardiac assessment as fetal echocardiography.

** For the purposes of analysis, studies were divided into five groups based on the protocol they used for cardiac assessment: (1) no protocol used, (2) Assessment of four chamber view without use of Colour Flow Doppler, (3) Assessment of four chamber view with use of Colour Flow Doppler, (4) Assessment of four chamber view and any type of outflow tract evaluation without Colour Flow Doppler, (5) Assessment of four chamber view and any type of outflow tract evaluation with use of Colour Flow Doppler.

^Evaluation of the cardiac outflow and inflow tracts varied significantly and assessment is listed in the table as described by each study respectively. 3VV – Three vessel view. 3VVT - Three-vessel and trachea view.

**Table S4** Details of anatomical protocols used by studies evaluating high-risk populations

| Study | No Protocol Used | Situs | Cardiac Axis | Four Chamber View | Outflow/Inflow Tract Assessment^ | Colour Flow Doppler | Pulsed Doppler | Ductus Venosus Assessment | Tricuspid Valve Assessment | Fetal ECHO* | Protocol Analysis Group** |
| --- | --- | --- | --- | --- | --- | --- | --- | --- | --- | --- | --- |
| Carvalho 1998^16^ | x | ✓ | x | ✓ | Visualization of two separate great vessels | x | X | x | x | ✓ | 4 |
| Zosmer  1999^75^ | x | ✓ | ✓ | ✓ | Outflow tracts; venous return | ✓ | ✓ | x | x | ✓ | 5 |
| Comas Gabriel 2002^76^ | x | x | x | ✓ | Origin and crossing of the great arteries; aortic and ductal arches; systemic venous return | ✓ | ✓ | x | x | ✓ | 5 |
| Den Hollander 2002^77^ | x | x | x | ✓ | Connection of the great arteries | x | X | x | x | x | 4 |
| Haak  2002^78^ | x | ✓ | x | ✓ | Long axis aorta view, crossing of the outflow tracts, longitudinal view aortic arch | x | X | x | x | ✓ | 4 |
| Huggon 2002^79^ | x | x | x | ✓ | Outflow tracts | ✓ | ✓ | x | ✓ | ✓ | 5 |
| Weiner  2002^80^ | x | x | x | ✓ | Long axis of the aorta, short axis of great vessels, aortic and ductal arch, IVC, SVC, Vena pulmonalis connections. | ✓ | ✓ | ✓ | ✓ | ✓ | 5 |
| Chen  2004^81^ | x | ✓ | x | ✓ | Outflow tracts | x | X | x | x | x | 4 |
| Bronshtein 2008^82^ | x | x | x | ✓ | Outlet vessels | x | X | x | x | x | 4 |
| Weiner  2008^83^ | x | x | x | ✓ | Short axis of great vessels, aortic and ductal arches, venous connection, AV valves and great vessels. | ✓ | ✓ | ✓ | ✓ | ✓ | 5 |
| Persico  2011^84^ | x | ✓ | ✓ | ✓ | Crossing of the aorta and the main pulmonary artery, aortic arch ductus arteriosus | ✓ | ✓ | x | ✓ | ✓ | 5 |
| Volpe  2012^85^ | x | ✓ | x | ✓ | Right and left outflow tracts, crossover of the great arteries, 3VVT | ✓ | X | x | ✓ | ✓ | 5 |
| Votino  2012^86^ | x | ✓ | x | ✓ | Left and Right outflow tracts, Aortic arch, ductus arteriosus, systemic veins, pulmonary veins. | x | X | x | x | x | 4 |
| Miller  2013^87^ | x | x | x | ✓ | X | x | ✓ | ✓ | x | x | 2 |
| Zidere  2013^88^ | x | x | x | ✓ | Outflow Tracts | ✓ | ✓ | x | x | ✓ | 5 |
| D'Antonio 2016^109^ | ✓ | x | x | x | X | x | X | x | x | x | 1 |
| Zalel  2017^90^ | x | ✓ | x | ✓ | Great vessels, outflow tracts, pulmonary and systemic venous return | x | X | x | x | x | 4 |
| Syngelaki  2020^91^ | Protocol evolved and changed over the course of the study period, therefore unable to include in analysis | | | | | | | | | |  |

(✓) Identifies anatomical views and/or examinations included in the study protocol.

(x) Identifies anatomical views and/or examinations which were not routinely reported as being included in the study protocol.

*Identifies studies which have described their first trimester cardiac assessment as fetal echocardiography.

** For the purposes of analysis, studies were divided into five groups based on the protocol they used for cardiac assessment: (1) no protocol used, (2) Assessment of four chamber view without use of Colour Flow Doppler, (3) Assessment of four chamber view with use of Colour Flow Doppler, (4) Assessment of four chamber view and any type of outflow tract evaluation without Colour Flow Doppler, (5) Assessment of four chamber view and any type of outflow tract evaluation with use of Colour Flow Doppler.

^Evaluation of the cardiac outflow and inflow tracts varied significantly and assessment is listed in the table as described by each study respectively. 3VVT - Three-vessel and trachea view. IVC – Inferior Vena Cava. SVC – Superior Vena Cava.

**Table S5** Number of major cardiac anomalies diagnosed or suspected in the first trimester with independent secondary confirmation, in non-high-risk populations

| Study | Major Cardiac Anomalies Detected or Suspected  In First Trimester  (TP - n) | False Positive Diagnoses following Diagnosed or Suspected Major Cardiac Anomaly in First Trimester  (FP - n) | Secondary Confirmation of T1 Detected Anomalies |
| --- | --- | --- | --- |
|  |  |  | Major Cardiac Anomalies (TP) with post-mortem or postnatal confirmation, n (%) |
| Whitlow 1999^31^ | 2 | 0 | NR |
| Michailidis 2001^32^ | 1 | 0 | NR |
| McAuliffe 2005^33^ | 0 | 0 | NA |
| Cedergren 2006^34^ | 0 | 0 | NA |
| Souka 2006^35^ | 3 | 0 | 2 (66.67%) |
| Srisupundit 2006^36^ | 2 | 0 | NR |
| Vimpelli 2006^37^ | 1 | 0 | NR |
| Dane 2007^38^ | 1 | 0 | NR |
| Lombardi 2007^39^ | 3 | 0 | 2 (66.67%) |
| Chen 2008^40^ (control group) | 1 | 0 | 1 (100.00%) |
| Chen 2008^40^ (study group) | 6 | 0 | 6 (100.00%) |
| Li 2008^41^ | 1 | 0 | NR |
| Bennasar 2009^42^ | 10 | 0 | 10 (100.00%) |
| Oztekin 2009^43^ | 0 | 0 | NA |
| Sinkovskaya 2010^45^ | 6 | 0 | 6 (100.00%) |
| Hartge 2011^46^ | 85 | 0 | 50 (58.82%) |
| Jakobsen 2011^47^ | 3 | 0 | NR |
| Krapp 2011^48^ | 17 | 0 | 6 (35.29%) |
| Syngelaki 2011^17^ | 29 | 0 | NR |
| Volpe 2011^49^ | 21 | 0 | 15 (71.43%) |
| Becker 2012^50^ | 7 | 0 | NR |
| Eleftheriades 2012^51^ | 16 | 0 | 1 (6.25%) |
| Grande 2012^52^ | 24 | 0 | NR |
| Novotna 2012^53^ | 1 | 0 | NR |
| Pilalis 2012^54^ | 2 | 0 | NR |
| Iliescu 2013^55^ | 32 | 0 | NR |
| Wang 2013^56^ | 4 | 0 | NR |
| Orlandi 2014^57^ | 19 | 0 | 3 (15.79%) |
| Andrew2015^58^ | 3 | 0 | NR |
| Colosi 2015^59^ | 0 | 0 | NA |
| Takita 2016^61^ | 2 | 0 | NR |
| Tudorache 2016^62^ | 25 | 14 | 6 (24.00%) |
| Vellamkondu 2017^64^ | 1 | 0 | 1 (100.00%) |
| Fernandez 2018^65^ | 5 | 0 | 5 (100.00%) |
| Kenkhuis 2018^66^ | 5 | 0 | 2 (40.00%) |
| Sainz 2018^67^ | 13 | 0 | NR |
| Vayna 2018^68^ | 22 | 0 | 6 (27.27%) |
| Zheng 2018^69^ | 28 | 0 | 28 (100.00%) |
| Chen 2019^70^ | 63 | 3 | NR |
| Duta 2019^71^ | 26 | 0 | NR |
| Ebrashy 2019^72^ | 85 | 16 | NR |
| Erenel 2019^73^ | 12 | 2 | 5 (41.67%) |
| Syngelaki 2019^74^ | 112 | 0 | NR |
| Pooled Result | **699** | **35** | **155 (22.17%)** |

TP – True Positive, FP – False Positive, NR – Not reported by study, NA – Not applicable.

**Table S6** Number of major cardiac anomalies diagnosed or suspected in the first trimester with independent secondary confirmation, in high-risk populations

| Study | Major Cardiac Anomalies Detected or Suspected  In First Trimester  (TP - n) | False Positive Diagnoses following Diagnosed or Suspected Major Cardiac Anomaly in First Trimester  (FP - n) | Secondary Confirmation of T1 Detected Anomalies |
| --- | --- | --- | --- |
|  |  |  | Major Cardiac Anomalies (TP) with post-mortem or postnatal confirmation  n (%) |
| Carvalho 1998^16^ | 2 | 0 | 2 (100.00%) |
| Zosmer 1999^75^ | 6 | 0 | 3 (37.50%) |
| Comas Gabriel 2002^76^ | 23 | 0 | 9 (39.13%) |
| Den Hollander 2002^77^ | 2 | 0 | 2 (100.00%) |
| Haak 2002^78^ | 9 | 1 | 7 (77.78%) |
| Huggon 2002^79^ | 65 | 2 | 28 (43.08%) |
| Weiner 2002^80^ | 6 | 0 | NR |
| Chen 2004^81^ | 8 | 0 | 4 (50.00%) |
| Bronshtein 2008^82^ | 8 | 0 | 0 (0.00%) |
| Weiner 2008^83^ | 13 | 5 | 11 (100.00%) |
| Persico 2011^84^ | 50 | 0 | 5 (10.42%) |
| Volpe 2012^85^ | 42 | 5 | NA |
| Votino 2012^86^ | 2 | 0 | NR |
| Miller 2013^87^ | 0 | 0 | NA |
| Zidere 2013^88^ | 52 | 5 | NR |
| D'Antonio 2016^109^ | 2 | 0 | 2 (100.00%) |
| Zalel 2017^90^ | 27 | 0 | 0 (0.00%) |
| Syngelaki 2020^91^ | 18 | 0 | NR |
| **Pooled Result** | **335** | **18** | **73 (21.79%)** |

TP – True Positive, FP – False Positive, NR – Not reported by study, NA – Not applicable.

**Table S7** Impact of first-trimester imaging protocol on the detection of major cardiac anomalies in high-risk populations

| Anatomical Protocol Used: | No Formal  Protocol* | 4-CV  (No CF Doppler) | 4-CV  + CF Doppler | 4-CV  + any view of outflow tracts  (No CF Doppler) | 4-CV  + any view of outflow tracts + CF Doppler |
| --- | --- | --- | --- | --- | --- |
| Number of Studies (n) | 1 | 1 | - | 7 | 8 |
| Number of Fetuses (n) | 2128 | 293 | - | 1851 | 4338 |
| Pooled Sensitivity, % (95% C.I) | **16.67**  (2.09 – 48.41) | **0.00**  (0.00 – 0.41) | **-** | **78.13**  (53.25 – 95.22) | **76.84**  (69.78 – 83.23) |

This table demonstrates results from a sub-group analysis performed based on the type of anatomical protocol used for first trimester cardiac screening by each study. Studies were categorized into one of four possible sub-groups based on protocol. A chi-squared test (2 by k) comparing all 4 protocols showed statistical significance difference (P<0.0001) with a chi-square test for linear trend suggesting a statistically significant (P<0.0001) increase in screening sensitivity with increasing level of detail in the anatomical protocol used. *No formal protocol indicates either studies without a dedicated ultrasound checklist or a protocol without a dedicated cardiac assessment. This table includes only studies with protocols available for analysis (see Table S4).

**Table S8** Characteristics of major cardiac anomalies diagnosed following first-trimester ultrasound assessment in non-high-risk populations

| Study | Total Major Cardiac Anomalies Present  (TP + FN) | Major Cardiac Anomalies Detected  In First Trimester  (TP)  (n) | FP  (n) | T1 Cardiac Anomalies with change of diagnosis at later gestation  (n) | Sensitivity for Detection of Major Cardiac Anomalies  (% - 95% C.I.) | % of Antenatal Major Cardiac Anomaly Diagnoses made in the first trimester  (% - 95% C.I.) | Specificity | PPV |
| --- | --- | --- | --- | --- | --- | --- | --- | --- |
| Whitlow 1999^31^ | 12 | 2 | 0 | 0 | 16.67  (2.09 – 48.41) | 22.22  (2.81 – 60.01) | 100.00  (99.94 - 100.00) | 100.00  (15.81 – 100.00) |
| Michailidis 2001^32^ | 10 | 1 | 0 | 0 | 10.00  (0.25 – 44.50) | 20.00  (0.51 – 71.64) | 100.00  (99.94 - 100.00) | 100.00  (2.50 – 100.00) |
| McAuliffe 2005^33^ | 2 | 0 | 0 | 0 | 0.00  (0.00-84.19) | 0.00  (0.00 – 97.50) | 99.85  (98.56 - 100.00) | 50.00  (0.04 - 99.96) |
| Cedergren 2006^34^ | 3 | 0 | 0 | 0 | 0.00  (0.00-70.76) | 0.00  (0.00- 99.94) | 99.98  (99.82 – 100.00) | 50.00  (0.04 - 99.96) |
| Souka 2006^35^ | 4 | 0 | 0 | 0 | 0.00  (0.00 – 60.24) | 0.00  (0.00 – 60.24) | 100.00  (99.83-100.00) | 100.00  (90.00 – 100.00) |
| Srisupundit 2006^36^ | 2 | 2 | 0 | 0 | 100.00  (15.81 – 100.00) | 100.00  (15.81 – 100.00) | 100.00  (99.38 – 100.00) | 100.00  (15.81 – 100.00) |
| Vimpelli 2006^37^ | 6 | 1 | 0 | 0 | 16.67  (0.42 – 64.12) | 50.00  (1.26 – 98.74) | 100.00  (99.36 – 100.00) | 100.00  (2.50 – 100.00) |
| Dane 2007^38^ | 4 | 1 | 0 | 0 | 25.00  (0.63 – 80.59) | 33.33  (0.84 – 90.57) | 100.00  (99.71 – 100.00) | 100.00  (2.50 – 100.00) |
| Lombardi 2007^39^ | 3 | 0 | 0 | 0 | 0.00  (0.00 – 70.76) | 16.67  (0.00 – 82.33) | 99.92  (99.25 - 100.00 ) | 50.00  (0.04 - 99.96) |
| Chen 2008^40^  (control group) | 18 | 1 | 0 | 0 | 5.56  (0.14 – 27.29) | 10.00  (0.25 – 44.50) | 100.00  (99.90 – 100.00) | 100.00  (2.50 – 100.00) |
| Chen 2008^40^  (study group) | 17 | 5 | 0 | 1 | 35.29  (14.21 – 61.67) | 42.86  (17.66 – 71.14) | 100.00  (99.91 – 100.00) | 100.00  (54.07 – 100.00) |
| Li 2008^41^ | 5 | 1 | 0 | 0 | 20.00  (0.51 – 71.64) | 33.33  (0.84 – 90.57) | 100.00  (99.83 – 100.00) | 100.00  (2.50 – 100.00) |
| Bennasar 2009^42^ | 11 | 10 | 0 | 0 | 90.91  (58.72 – 99.77) | 100.00  (69.15 – 100.00) | 100.00  (93.28 – 100.00) | 100.00  (69.15 – 100.00) |
| Oztekin 2009^43^ | 3 | 0 | 0 | 0 | 0.00  (0.00 – 70.76) | 0.00  (0.00 – 97.50) | 99.95  (99.57 – 100.00) | 50.00  (0.04 - 99.96) |
| Sinkovskaya 2010^45^ | 8 | 6 | 0 | 0 | 75.00  (34.91 – 96.81) | 75.00  (34.91 – 96.81) | 100.00  (96.07 – 100.00) | 100.00  (54.07 – 100.00) |
| Hartge 2011^46^ | 101 | 85 | 0 | 0 | 84.16  (75.55-90.67) | 85.86  (77.41 – 92.05) | 100.00  (99.89 – 100.00) | 100.00  (95.75 – 100.00) |
| Jakobsen 2011^47^ | 43 | 3 | 0 | 0 | 6.98  (1.46 – 19.06) | 25.00  (5.49 – 57.19) | 100.00  (99.96 – 100.00) | 100.00  (29.24 – 100.00) |
| Krapp 2011^48^ | 19 | 17 | 0 | 0 | 89.47  (66.86 – 98.70) | 89.47  (66.86 – 98.70) | 100.00  (99.45 – 100.00) | 100.00  (80.49 – 100.00) |
| Syngelaki 2011^17^ | 115 | 29 | 0 | 0 | 25.22  (17.58 – 34.17) | 26.61  (18.60 – 35.92) | 100.00  (99.99 – 100.00) | 100.00  (88.06 – 100.00) |
| Volpe 2011^49^ | 26 | 17 | 0 | 4 | 80.77  (60.65 – 93.45) | 80.77  (60.65 – 93.45) | 100.00  (99.92 – 100.00) | 100.00  (83.89 – 100.00) |
| Becker 2012^50^ | 15 | 7 | 0 | 0 | 46.67  (21.27 – 73.41) | 58.33  (27.67 – 84.83) | 100.00  (99.94-100.00) | 100.00  (59.04 -100.00) |
| Eleftheriades 2012^51^ | 34 | 16 | 0 | 0 | 47.06  (29.78 – 64.87) | 48.48  (30.80 – 66.46) | 100.00  (99.90 – 100.00) | 100.00  (79.41 – 100.00) |
| Grande 2012^52^ | 37 | 24 | 0 | 0 | 64.86  (47.46 - 79.79) | 68.57  (50.71 – 83.15) | 100.00  (99.97 – 100.00) | 100.00  (85.75 – 100.00) |
| Novotna 2012^53^ | 18 | 1 | 0 | 0 | 5.56  (0.14 - 27.29) | 14.29  (0.36 – 57.87) | 100.00  (99.96 -100.00) | 100.00  (2.50 – 100.00) |
| Pilalis 2012^54^ | 11 | 2 | 0 | 0 | 18.18  (2.28 – 51.76) | 18.18  (2.28 – 51.78) | 100.00  (99.91 -100.00) | 100.00  (15.81 – 100.00) |
| Iliescu 2013^55^ | 34 | 29 | 0 | Unable to Report** | 85.29  (68.94 – 95.05) | 85.29  (68.94 – 95.05) | 100.00  (99.93 – 100.00) | 100.00  (88.06 – 100.00) |
| Wang 2013^56^ | 10 | 4 | 0 | 0 | 40.00  (12.16 – 73.76) | 44.44  (13.70 – 78.80) | 100.00  (99.87 – 100.00) | 100.00  (39.76 – 100.00) |
| Orlandi 2014^57^ | 21 | 16 | 0 | 1 | 89.47  (66.86 – 98.70) | 85.00  (62.11 – 96.79) | 100.00  (99.92 – 100.00) | 100.00  (80.49 – 100.00) |
| Andrew2015^58^ | 3 | 1 | 0 | 0 | 33.33  (0.84 – 90.57) | 33.33  (0.84 – 90.57) | 100.00  (99.92 – 100.00) | 100.00  (80.49 – 100.00) |
| Colosi 2015^59^ | 3 | 0 | 0 | 0 | 0.00  (0.00 – 70.76) | 0.00  (0.00 – 70.76) | 100.00  (99.92 – 100.00) | 100.00  (2.50 – 100.00) |
| Takita 2016^61^ | 15 | 2 | 0 | 0 | 13.33  (1.66 – 40.46) | 33.33  (4.33 – 77.72) | 99.99  (99.92 – 100.00) | 50.00  (0.04 -99.96) |
| Tudorache 2016^62^ | 32 | 22 | 13 | 1 | 75.00  (56.60 – 88.54) | 71.88  (53.25 – 86.25) | 100.00  (99.82 – 100.00) | 100.00  (15.81 – 100.00) |
| Vellamkondu 2017^64^ | 4 | 1 | 0 | 0 | 25.00  (0.63 – 80.59) | 50.00  (1.26 – 98.74) | 99.60  (99.31 - 99.78) | 63.89  (46.22 - 79.18) |
| Fernandez 2018^65^ | 5 | 5 | 0 | 0 | 100.00  (47.82- 100.00) | 100.00  (47.81 – 100.00) | 100.00  (99.16 – 100.00) | 100.00  (2.50 – 100.00) |
| Kenkhuis 2018^66^ | 13 | 5 | 0 | 0 | 38.46  (13.86 – 68.42) | 50.00  (18.71 – 81.29) | 100.00  (99.44 – 100.00) | 100.00  (47.82 – 100.00) |
| Sainz 2018^67^ | 15 | 13 | 0 | 0 | 86.67  (59.54 – 98.34) | 86.67  (59.54 – 98.34) | 100.00  (99.93 – 100.00) | 100.00  (47.82 – 100.00) |
| Vayna 2018^68^ | 31 | 22 | 0 | 0 | 70.97  (51.96 – 85.78) | 75.86  (56.46 – 89.70) | 100.00  (99.25 – 100.00) | 100.00  (75.29 – 100.00) |
| Zheng 2018^69^ | 30 | 27 | 0 | (1)* | 93.33  (77.93 – 99.18) | 93.33  (77.93 – 99.18) | 100.00  (99.94 – 100.00) | 100.00  (84.56 – 100.00) |
| Chen 2019^70^ | 121 | 63 | 0 | 0 | 52.07  (42.80 – 61.23) | 52.07  (42.80 – 61.23) | 100.00  (99.76 – 100.00) | 100.00  (87.66 – 100.00) |
| Duta 2019^71^ | 34 | 26 | 0 | 0 | 76.47  (58.82 – 89.25) | 81.25  (63.56 – 92.79) | 100.00  (99.96 – 100.00) | 100.00  (94.31 – 100.00) |
| Ebrashy 2019^72^ | 100 | 85 | 0 | 0 | 85.00  (76.47 – 91.35) | 85.00  (76.47 – 91.35) | 100.00  (99.95 – 100.00) | 100.00  (86.77 – 100.00) |
| Erenel 2019^73^ | 12 | 10 | 2 | 1 | 91.67  (61.52 – 99.79) | 91.67  (61.52 – 99.79) | 99.71  (98.97 – 99.97) | 84.62  (54.55 – 98.08) |
| Syngelaki 2019^74^ | 354 | 112 | 0 | 0 | 31.64  (26.82 – 36.76) | 32.94  (27.97 – 38.22) | 100.00  (99.89 – 100.00) | 100.00  (95.75 – 100.00) |
| **Pooled**  **Result** | **1364** | **674** | **15** | **9** | **51.20**  **(40.92 – 61.43)** | **57.81**  **(47.48 – 66.30)** | **99.99**   - 1. **– 100.00)** | **96.58**  **(93.95– 98.48)** |

*1 x case described by study authors as resolution of ventricular aneurysm

** Iliescu et al. (2013) report 108 false positive results relating to the cardiovascular system, but no breakdown which would allow understanding of what proportion of these anomalies constitute false positive of major cardiac anomalies and therefore this could not be reported. FN – False negative. FP – False positive. NA – Not applicable. NR – Not reported. TP – True Positive.

**Table S9** Characteristics of major cardiac anomalies suspected following first-trimester ultrasound assessment in non-high-risk populations

| Study | Sample Size  (n) | Total Major Cardiac Anomalies Present in Study Population  (n) | Number of Suspected Diagnoses made in T1 | Suspected Diagnosis Confirmed*  (TP - n) | FP  (n) | Major Cardiac Anomaly Confirmed, but Change in Specific Diagnosis  (n) | Sensitivity of Suspected Diagnosis for the Detection of Major Cardiac Anomalies  (% - 95% C.I.)^ | Specificity of Suspected Diagnosis for the Detection of Major Cardiac Anomalies  (% - 95% C.I) | Positive Predictive Value of Suspected Diagnosis in the Detection of Major Cardiac Anomalies  (95% C.I.) |
| --- | --- | --- | --- | --- | --- | --- | --- | --- | --- |
| Souka 2006^35^ | 1148 | 4 | 3 | 3 | 0 | 0 | 75.00  (19.41 – 99.37) | 100.00  (99.68 – 100.00) | 100.00  (29.24 – 100.00) |
| Lombardi 2007^39^ | 623 | 3 | 3 | 3 | 0 | 0 | 100.00  (29.24 – 100.00) | 100.00  (99.41 – 100.00) | 100.00  (29.24 – 100.00) |
| Iliescu 2013^55^ | 5472 | 34 | 3 | 3 | 0 | 0 | 60.00  (14.66 – 94.73) | 100.00  (99.93 – 100.00) | 100.00  (29.24 – 100.00) |
| Orlandi 2014^57^ | 4820 | 21 | 2 | 2 | 0 | 0 | 50.00  (6.76 – 93.24) | 100.00  (99.92 – 100.00) | 100.00  (15.81 – 100.00) |
| Andrew2015^58^ | 4421 | 3 | 2 | 2 | 0 | 0 | 100.00  (15.81 – 100.00) | 100.00  (99.92 – 100.00) | 100.00  (15.81 – 100.00) |
| Tudorache 2016^62^ | 3240 | 32 | 3 | 2 | 1 | 0 | 22.22  (2.81 – 60.00) | 99.97  (99.83 – 99.99) | 66.67  (9.43 – 99.16) |
| Chen 2019^70^ | 10294 | 121 | 3 | 0 | 3 | 0 | 0.00  (0.00 – 6.16) | 99.97  (99.91 – 99.99) | 0.00  (0.00 – 70.76) |
| Ebrashy 2019^72^ | 3400 | 100 | 16 | 0 | 16 | 0 | 0.00  (0.00 – 21.80) | 99.52  (99.22 – 99.72) | 0.00  (0.00 – 20.59) |
| Erenel 2019^73^ | 707 | 12 | 1 | 1 | 0 | 1 | 100.00  (2.50 – 100.00) | 100.00  (99.47 – 100.00) | 100.00  (2.50 – 100.00) |
| Pooled | 34,125 | 330 | 36 | 16 | 20 | 1 | 44.60  (15.08 – 76.41) | 99.96  (99.88 – 100.00) | 67.81  (27.84 – 96.37) |

*Refers to a diagnosis which was confirmed either on ultrasound at later gestation, on post-mortem and/or postnatally and therefore considered a true positive. False negative in this situation was considered number of anomalies not diagnosed, suspected or labelled as AUS at time of the first trimester scan. ^For the purposes of this sensitivity calculation, an anomaly which was suspected in the first trimester but underwent a subsequent change in diagnosis, was a considered a true positive for a major cardiac anomaly.

**Table S10** Characteristics of major cardiac anomalies reported as cardiac abnormality of unknown significance (AUS) following first-trimester ultrasound assessment in non-high-risk populations

| Study | Sample Size  (n) | Total Major Cardiac Anomalies Present in Study Population  (n) | Number of AUS Diagnoses Given in T1 | TP  (n) | FP  (n) | Sensitivity of AUS for the Detection of Major Cardiac Anomalies  (% - 95% C.I.) | Specificity of AUS for the Detection of Major Cardiac Anomalies  (% - 95% C.I) | Positive Predictive Value of AUS in the Detection of Major Cardiac Anomalies  (95% C.I.) |
| --- | --- | --- | --- | --- | --- | --- | --- | --- |
| Abu Rustum 2010^44^* | 1370 | 11 | 10 | 9 | 1 | 81.89  (48.22 – 97.72) | 99.93  (99.59 – 100.00) | 90.00  (55.50 – 99.75) |
| Wiechec 2015^60^* | 1084 | 37 | 33 | 33 | 0 | 89.19  (74.58 – 96.97) | 100.00  (99.65 – 100.00) | 100.00  (89.42 – 100.00) |
| De Robertis 2017^63^* | 5343 | 33 | 32 | 26 | 6 | 78.79  (61.09 – 91.02) | 99.89  (99.75 – 99.96) | 81.25  (63.56 -92.79) |
| Kenkhuis 2018^66^ | 5534 | 13 | 1 | 0 | 1 | 0.00  (0.00 – 36.94) | 99.98  (99.89 – 99.99) | 0.00  (0.00 – 97.50) |
| Pooled | 13,331 | 94 | 76 | 68 | 8 | 63.00  (28.53 – 91.24) | 99.94  (99.87 – 99.98) | 85.95  (61.48 – 99.03) |

*Studies screened exclusively for abnormalities of the four chamber or outflow tract views in the first trimester (eg. ventricular and/or outflow tract disproportions, abnormalities in spatial relationship of vessels, etc.) with the objective of providing a formal and specific diagnosis at a later gestation in pregnancy.

**Table S11** Details of cases in which diagnosis or suspicion of a specific major cardiac anomaly made in the first trimester was changed, in non-high-risk populations

| **Study**  **(Year)** | **First Trimester Diagnosis** | **Second Trimester Diagnosis** | **Postnatal/Post mortem Confirmation** |
| --- | --- | --- | --- |
| Chen 2008^40^ | VSD | Complex Heart Disease | Confirmed |
| Volpe 2011^49^ | VSD | Partial AVSD | Confirmed |
| Volpe 2011^49^ | DORV | TGA | Confirmed |
| Volpe 2011^49^ | Critical Aortic Stenosis | HLHS | Unclear |
| Volpe 2011^49^ | Misaligned VSD | TOF | Confirmed |
| Orlandi 2014^57^ | Single Ventricle + Truncus Arteriosus | Single Ventricle + DORV | TOP –  Unconfirmed by autopsy |
| Tudorache 2016^62^ | HRHS | Tricuspid atresia with intact septum | TOP –  Unconfirmed by autopsy |
| Erenel 2019^73^ | Suspected HLHS | Coarctation of the aorta with right to left ventricular disproportion | Confirmed |
| Erenel 2019^73^ | Tetralogy of Fallot and pulmonary valve regurgitation | Absent Pulmonary Valve Syndrome, Agenesis of ductus arteriosus, VSD, Over-riding aorta | Confirmed |

**Table S12** Characteristics of major cardiac anomalies diagnosed following first-trimester ultrasound assessment in high-risk populations

| Study | Prevalence of Major Cardiac Anomalies  (n per 100 fetuses) | Total Major Cardiac Anomalies Present  (TP + FN) | Major Cardiac Anomalies Detected  In First Trimester  (TP)  (n) | FP  (n) | T1 Cardiac Anomalies with change of diagnosis at later gestation  (n) | % Sensitivity for Detection of Major Cardiac Anomalies  (95% C.I.) | % of Antenatal Major Cardiac Anomaly Diagnoses made in the first trimester  (95% C.I.) | Specificity | PPV |
| --- | --- | --- | --- | --- | --- | --- | --- | --- | --- |
| Carvalho 1998^16^ | 26.67  (7.79 – 55.10) | 4 | 2 | 0 | 0 | 50.00  (6.76 – 93.24) | 100.00  (15.81 – 100.00) | 100.00  (71.51 – 100.00) | 100.00  (15.81 – 100.00) |
| Zosmer  1999^75^ | 8.93  (4.37 – 15.81) | 10 | 6 | 0 | 6 | 75.00  (34.91 – 96.81) | 75.00  (34.91 – 96.81) | 100.00  (96.45 – 100.00) | 100.00  (54.07 – 100.00) |
| Comas Gabriel 2002^76^ | 16.50  (11.64 – 22.38) | 33 | 23 | 0 | 0 | 71.88  (34.91 – 96.81) | 79.31  (60.28 – 92.01) | 100.00  (97.82 – 100.00) | 100.00  (85.18 – 100.00) |
| Den Hollander 2002^77^ | 2.97  (0.62 – 8.44) | 3 | 2 | 0 | 0 | 66.67  (9.43 – 99.16) | 66.67  (9.42 – 99.16) | 100.00  (96.31 – 100.00) | 100.00  (15.81 – 100.00) |
| Haak  2002^78^ | 35.56  (21.87 – 51.22) | 16 | 7 | 0 | 0 | 50.00  (23.04 – 76.96) | 100.00  (59.04 – 100.00) | 100.00  (88.06 – 100.00) | 100.00  (59.04 – 100.00) |
| Huggon 2002^79^ | 16.11  (12.93 – 19.72) | 77 | 61 | 0 | 0 | 83.56  (73.05 – 91.21) | 88.41  (78.43 – 94.86) | 100.00  (99.08 – 100.00) | 100.00  (94.13 – 100.00) |
| Weiner  2002^80^ | 1.79  (0.72 – 3.64) | 7 | 6 | 0 | 0 | 85.71  (42.13 – 99.64) | 85.71  (42.13 – 99.64) | 100.00  (99.05 – 100.00) | 100.00  (54.07 – 100.00) |
| Chen  2004^81^ | 0.87  (0.47 – 1.45) | 14 | 6 | 0 | 0 | 50.00  (21.09 – 78.91) | 75.00  (34.91 – 96.81) | 100.00  (99.77 – 100.00) | 100.00  (54.07 – 100.00) |
| Bronshtein 2008^82^ | 34.78  (16.38 – 57.27) | 8 | 8 | 0 | 0 | 100.00  (63.06 – 100.00) | 100.00  (63.06 – 100.00) | 100.00  (78.20 – 100.00) | 100.00  (63.06 – 100.00) |
| Weiner  2008^83^ | 11.00  (7.02 – 16.18) | 22 | 11 | 0 | 1 | 57.14  (34.02 – 78.18) | 57.14  (34.02 – 78.18) | 100.00  (97.95 – 100.00) | 100.00  (73.54 – 100.00) |
| Persico  2011^84^ | 6.32  (4.81 – 8.13) | 56 | 48 | 0 | 2 | 89.29  (78.12 – 95.97) | 89.29  (78.12 – 95.97) | 100.00  (99.56 – 100.00) | 100.00  (92.89 – 100.00) |
| Volpe  2012^85^ | 6.55  (5.00 – 8.41) | 57 | 35 | 5 | 7 | 73.68  (60.34 – 84.46) | 75.00  (61.63 – 85.61) | 99.39  (98.58 – 99.80) | 89.36  (76.90 – 96.45) |
| Votino  2012^86^ | 13.33  (1.66 – 40.46) | 2 | 0 | 0 | 0 | 50.00  (0.00 – 99.96) | 50.00  (0.00 – 99.96) | 100.00  (75.29 – 100.00) | 100.00  (0.06 – 100.00) |
| Miller  2013^87^ | 2.40  (0.97 – 4.86) | 7 | 0 | 0 | 0 | 0.00  (0.00 – 40.96) | 0.00  (0.00 – 45.93) | 100.00  (98.72 – 100.00) | 50.00  (0.04 – 99.96) |
| Zidere  2013^88^ | 6.08  (4.80 – 7.59) | 73 | 42 | 1 | 0 | 69.57  (57.31 – 80.06) | 71.64  (59.31 – 81.99) | 99.91  (99.51 – 100.00) | 97.96  (89.15 – 99.95) |
| D'Antonio 2016^109^ | 0.56  (0.29 – 0.98 | 12 | 2 | 0 | 0 | 16.67  (2.09 – 48.41) | 16.67  (2.09 – 48.41) | 100.00  (99.83 – 100.00) | 100.00  (15.81 – 100.00) |
| Zalel  2017^90^ | 62.79  (46.73 – 77.02) | 27 | 27 | 0 | 0 | 100.00  (87.23 – 100.00) | 100.00  (87.23 – 100.00) | 100.00  (79.41 – 100.00) | 100.00  (87.23 – 100.00) |
| Syngelaki  2020^91^ | 0.41  (0.31 – 0.54) | 52 | 18 | 0 | 0 | 34.61  (21.97 – 49.09) | 100.00  (81.47 – 100.00) | 100.00  (99.97 – 100.00) | 100.00  (81.47 – 100.00) |
| **Pooled** | **9.42**  **(6.04 – 13.47)** | **480** | **304** | **6** | **16** | **65.27**  **(48.39 – 73.78)** | **77.28**  **(66.14 – 86.75)** | **99.93**  **(99.84 – 99.98)** | **97.65**  **(95.76 – 98.99)** |

FN – False negative. FP – False positive. NA – Not applicable. NR – Not reported. TP – True Positive.

**Table S13** Characteristics of major cardiac anomalies suspected following first-trimester ultrasound assessment in high-risk populations

| Study | Sample Size  (n) | Total Major Cardiac Anomalies Present in Study Population  (n) | Number of Suspected Diagnoses made in T1 | Suspected Diagnosis Confirmed*  (n) | FP  (n) | Major Cardiac Anomaly Confirmed, but Change in Specific Diagnosis  (n) | Sensitivity of Suspected Diagnosis for the Detection of Major Cardiac Anomalies  (% - 95% C.I.) | Specificity of Suspected Diagnosis for the Detection of Major Cardiac Anomalies  (% - 95% C.I) | Positive Predictive Value of Suspected Diagnosis in the Detection of Major Cardiac Anomalies  (95% C.I.) |
| --- | --- | --- | --- | --- | --- | --- | --- | --- | --- |
| Haak  2002^78^ | 45 | 16 | 3 | 2 | 1 | 0 | 22.22  (2.81 – 60.00) | 96.67  (82.78 – 99.92) | 66.67  (9.43 – 99.16) |
| Huggon 2002^79^ | 478 | 77 | 6 | 4 | 2 | 0 | 25.00  (7.26 – 52.38) | 99.50  (98.22 – 99.94) | 66.67  (22.28 – 95.67) |
| Chen  2004^81^ | 1609 | 14 | 2 | 2 | 0 | 0 | 25.00  (3.19 – 65.09) | 100.00  (99.77 – 100.00) | 100.00  (15.81 – 100.00) |
| Weiner  2008^83^ | 200 | 22 | 6 | 0 | 5 | 1 | 10.00  (0.25 – 44.50) | 97.27  (93.74 – 99.11) | 20.00  (0.51 – 71.64) |
| Votino  2012^86^ | 15 | 2 | 2 | 2 | 0 | 0 | 100.00  (15.81 – 100.00) | 100.00  (75.29 – 100.00) | 100.00  (15.81 – 100.00) |
| Zidere  2013^88^ | 1200 | 73 | 8 | 2 | 4 | 2 | 16.00  (4.54 – 36.08) | 99.64  (99.10 – 99.90) | 57.14  (18.41 – 90.10) |
| **Pooled** | **3547** | **204** | **27** | **12** | **12** | **3** | **24.43**  **(13.21 – 37.79)** | **99.28**  **(98.17 – 99.88)** | **60.73**  **(40.41 – 79.29)** |

**Table S14** Characteristics of major cardiac anomalies reported as cardiac abnormality of unknown significance (AUS) following first-trimester ultrasound assessment in high-risk populations

| Study | Sample Size  (n) | Total Major Cardiac Anomalies Present in Study Population  (n) | Number of AUS Diagnoses Given in T1 | TP  (n) | FP  (n) | Sensitivity of AUS for the Detection of Major Cardiac Anomalies  (% - 95% C.I.) | Specificity of AUS for the Detection of Major Cardiac Anomalies  (% - 95% C.I) | Positive Predictive Value of AUS in the Detection of Major Cardiac Anomalies  (95% C.I.) |
| --- | --- | --- | --- | --- | --- | --- | --- | --- |
| Zosmer  1999^75^ | 112 | 10 | 2 | 2 | 0 | 50.00  (6.76 – 93.24) | 100.00  (96.45 – 100.00) | 100.00  (15.81 – 100.00) |
| Comas Gabriel 2002^76^ | 200 | 33 | 1 | 1 | 0 | 10.00  (0.25 – 44.50) | 100.00  (97.82 – 100.00) | 100.00  (2.50 – 100.00) |
| Bronshtein 2008^82^ | 23 | 8 | 1 | 0 | 1 | 0.00  (0.00 - 97.50) | 93.75  (69.77 – 99.84) | 0.00  (0.00 – 97.50) |
| Votino  2012^86^ | 870 | 57 | 1 | 0 | 1 | (0.00 – 21.80) | 99.88  (99.32 – 100.00) | 0.00  (0.00 – 97.50) |
| **Pooled** | **1205** | **108** | **5** | **3** | **2** | **13.37**  **(0.83 – 37.37)** | **99.73**  **(99.07 – 99.99)** | **55.79**  **(12.91 – 93.81)** |

**Table S15** Details of cases in which diagnosis or suspicion of a specific major cardiac anomaly made in the first trimester was changed, in high-risk populations

| **Author** | **First Trimester Diagnosis** | **Suspected Diagnosis at First Trimester** | **Second Trimester Diagnosis** | **Postnatal/**  **Post- mortem Confirmation** |
| --- | --- | --- | --- | --- |
| Weiner  2008^83^ | VSD | NA | TOF | PM Confirmed |
| Weiner  2008^83^ | NA | VSD | TOF | PN Confirmed |
| Zidere  2013^88^ | NA | AVSD | VSD | NA |
| Zidere  2013^88^ | NA | CoA | Persistent left SVC | NA |
| Zidere  2013^88^ | TGA | NA | TOF | NA |
| Zidere  2013^88^ | TOF | NA | TGA | NA |
| Zidere  2013^88^ | Complex CHD | NA | TOF | NA |
| Zidere  2013^88^ | Ectopia Cordis | NA | Pentalogy of Cantrell | NA |
| Zidere  2013^88^ | Tricuspid Regurgitation | NA | Severe Ebstein's anomaly, pulmonary atresia | NA |
| Zidere  2013^88^ | Tricuspid Regurgitation + Possible Pulmonary Stenosis | NA | Mild Ebstein's anomaly and pulmonary stenosis | NA |
| Persico  2011^84^ | AVSD | NA | DORV, PS | NA |
| Persico  2011^84^ | Complex CHD | NA | TOF + Multiple VSDs | PN Confirmed |
| Volpe  2012^85^ | Tricuspid Regurgitation | NA | Pulmonary atresia with intact septum | PN Confirmed |
| Volpe  2012^85^ | Mitral Valve Regurgitation | NA | Critical Aortic Stenosis | PN Confirmed |
| Volpe  2012^85^ | Critical aortic stenosis | NA | Hypoplastic Left Heart Syndrome | T2 Confirmation |
| Volpe  2012^85^ | Malalignment VSD | NA | TOF | T2 Confirmation |
| Volpe  2012^85^ | Tricuspid Regurgitation + VSD | NA | Mild Ebstein anomaly | T2 Confirmation |
| Volpe  2012^85^ | TGA | NA | DORV | T2 Confirmation |
| Volpe  2012^85^ | TOF | NA | DORV | T2 Confirmation |

**Table S16** Screening characteristics of ultrasound in the first trimester for the detection of individual cardiac anomalies in non-high-risk populations

| Anomaly | Low Risk/Mixed Risk/Unselected Population | | | | | | | Characteristics of T1 Detected Anomalies | | Secondary Confirmation of T1 Anomalies |
| --- | --- | --- | --- | --- | --- | --- | --- | --- | --- | --- |
|  | Studies  (n) | Total  Anomalies (n) | Anomalies Detected^  (TP – n) | FP**  (n) | Diagnosis Change at a later GA  (n) | Detection rate*  %  (95% C.I.) | Specificity for Anomaly Detection (%) with 95% C.I. | Additional Cardiac Anomalies* | Additional Non-Cardiac Anomalies* | Anomalies with post-mortem or postnatal confirmation*  n (%) |
| HLHS | 30 | 145 | 118 | 1 | 1 | 73.28  (59.86 – 84.82) | 100.00  (100.00 – 100.00) | 9 | 5 | 23  (19.49) |
| HRHS | 7 | 20 | 19 | 2 | 1 | 91.65  (77.23 – 99.21) | 99.99  (99.97 – 99.99) | 3 | 0 | 0  (0) |
| Univentricle | 15 | 17 | 13 | 0 | 0 | 71.21  (52.11 – 87.03) | 99.99  (99.98 – 99.99) | 2 | 0 | 2  (15.38) |
| TOF | 31 | 120 | 50 | 1 | 0 | 40.95  (30.16 – 52.20) | 100.00  (100.00 – 100.00) | 7 | 3 | 10  (20.00) |
| TGA | 26 | 84 | 35 | 3 | 0 | 45.05  (29.29 – 61.35) | 100.00  (100.00 – 100.00) | 8 | 1 | 15  (42.86) |
| CoA | 24 | 67 | 26 | 0 | 0 | 37.23  (23.96 – 51.56) | 100.00  (99.99 – 100.00) | 12 | 2 | 10  (38.46) |
| VSD | 36 | 360 | 53 | 23 | 1 | 23.92  (14.41 – 34.97) | 99.99  (99.98 – 99.99) | 24 | 10 | 20  (37.74) |
| AVSD | 32 | 209 | 171 | 1 | 0 | 77.24  (63.62 – 88.42) | 100.00  (100.00 – 100.00) | 11 | 21 | 48  (28.07) |
| ASD | 8 | 16 | 3 | 0 | 0 | 21.53  (6.78 – 41.66) | 100.00  (99.99 – 100.00) | 0 | 1 | 2  (66.67) |
| Truncus Arteriosus | 13 | 19 | 16 | 0 | 1 | 76.73  (58.94 – 90.62) | 100.00  (100.00 – 100.00) | 5 | 4 | 3  (18.75) |
| DORV | 15 | 34 | 22 | 0 | 0 | 63.11  (44.90 – 79.59) | 100.00  (99.99 – 100.00) | 7 | 0 | 6  (27.27) |
| Heterotaxy Syndromes | 17 | 33 | 25 | 0 | 0 | 72.59  (55.75 – 86.63) | 100.00  (100.00 – 100.00) | 9 | 0 | 1  (3.03) |
| Ectopia Cordis | 5 | 13 | 13 | 0 | 0 | 93.26  (76.03 – 99.98) | 100.00  (100.00 – 100.00) | 0 | 9 | 5  (38.46) |
| Ebstein’s Anomaly | 7 | 11 | 2 | 0 | 0 | 25.03  (4.83 – 54.08) | 100.00  (99.99 – 100.00) | 0 | 0 | 1  (50.00) |
| Rhabdo-myoma | 3 | 12 | 0 | 0 | 0 | 4.87  (0.19 – 22.09) | 100.00  (100.00 – 100.00) | - | - | - |
| Aortic Stenosis | 10 | 24 | 9 | 3 | 1 | 38.81  (15.77 – 64.90) | 99.99  (99.98 – 99.99) | 6 | 1 | 2  (22.22) |
| Pulmonary valve/  Pulmonary Artery Stenosis | 15 | 42 | 7 | 0 | 0 | 19.45  (8.99 – 32.74) | 100.00  (100.00 – 100.00) | 3 | 0 | 3  (42.86) |
| Pulmonary Atresia | 10 | 26 | 17 | 0 | 0 | 59.68  (23.63 – 90.53) | 100.00  (100.00 – 100.00) | 5 | 1 | 3  (17.65) |
| Tricuspid Atresia/  Dysplasia | 12 | 28 | 26 | 2 | 0 | 88.63  (76.00 – 96.94) | 100.00  (99.99 – 100.00) | 3 | 1 | 3  (11.54) |
| Complex Cardiac Defects | 10 | 57 | 43 | 0 | 0 | 76.31  (57.46 – 90.92) | 100.00  (100.00 – 100.00) | - | 0 | 9  (20.93) |

^Refers to anomalies which were either diagnosed, suspected or labelled as AUS at time of first trimester ultrasound screening.

*Rates for individual anomalies were only calculated for those conditions where more than 10 cases were reported (see methods). This excluded the following abnormalities: Cardiomegaly (n = 7), Double Inlet Left Ventricle (n=6), Cardiomyopathy (n = 5), Ventricular Aneurysm (n=4), Endocardial Fibroelastosis (n=4), Aortic Arch Hypoplasia (n = 3), Total Anomolous Pulmonary Venous Drainage (n=3), Interrupted Aortic Arch (n = 2), Pulmonary Valve Regurgitation (n=2), Aortic Valve Atresia (n = 3), Mitral Valve Atresia (n=3), Polyvalvular Dysplasia (n=1), Cord Triatriatum (n=1). Detailed data available on request.

**The False positive (FP) rate used in the specificity calculation in this table includes those listed as FP plus those where the diagnosis was changed to another cardiac anomaly at a later gestation.

**Table S17** Screening characteristics of ultrasound in the first trimester for the detection of individual cardiac anomalies in high-risk populations

| Anomaly | High Risk Population | | | | | | | Characteristics of T1 Detected Anomalies | | Secondary Confirmation of T1 Anomalies |
| --- | --- | --- | --- | --- | --- | --- | --- | --- | --- | --- |
|  | Studies  (n) | Total  Anomalies (n) | Anomalies Detected in T1  (TP – n) | FP**  (n) | Diagnosis Change at a later GA  (n) | Detection rate*  %  (95% C.I.) | Specificity for Anomaly Detection (%) with 95% C.I. | Additional Cardiac Anomalies* | Additional Non-Cardiac Anomalies* | Anomalies with post-mortem or postnatal confirmation*  n (%) |
| HLHS | 11 | 32 | 24 | 0 | 0 | 69.29  (43.68 – 89.75) | 99.97  (99.91 – 100.00) | 4 | 4 | 8 (33.33) |
| TOF | 7 | 44 | 27 | 0 | 2 | 53.66  (32.89 – 73.78) | 99.90  (99.78 – 99.98) | 7 | 2 | 6 (22.22) |
| TGA | 9 | 21 | 17 | 0 | 2 | 81.48  (62.56 – 94.74) | 99.98  (99.93– 100.00) | 4 | 0 | 9 (52.94) |
| CoA | 8 | 33 | 21 | 3 | 1 | 59.88  (43.30 – 75.39) | 99.91  (99.79 – 99.98) | 5 | 1 | 9 (42.86) |
| VSD | 13 | 94 | 52 | 13 | 12 | 47.50  (26.75 – 68.70) | 99.64  (99.30 – 99.86) | 23 | 17 | 21 (40.38) |
| AVSD | 13 | 93 | 83 | 5 | 2 | 83.62  (69.56 – 93.93) | 99.87  (99.75 – 99.95) | 18 | 10 | 21 (25.30) |
| ASD | 7 | 11 | 1 | 0 | 0 | 16.11  (2.40 – 38.60) | 99.97  (99.91 – 100.00) | 6 | 2 | 1 (100.00) |
| DORV | 8 | 12 | 7 | 0 | 0 | 56.99  (27.99 – 83.58) | 99.97  (99.91 – 100.00) | 7 | 2 | 0 (0.00) |
| Heterotaxy Syndromes | 8 | 16 | 14 | 0 | 0 | 83.05  (64.00 – 95.85) | 99.97  (99.92 – 100.00) | 10 | 2 | 5 (35.71) |
| Pulmonary valve/  Pulmonary Artery Stenosis | 7 | 16 | 5 | 0 | 0 | 29.12  (7.50 – 57.68) | 99.97  (99.92 – 100.00) | 3 | 1 | 3 (60.00) |
| Pulmonary Atresia | 7 | 12 | 8 | 0 | 0 | 65.26  (38.77 – 87.45) | 99.97  (99.90 – 100.00) | 8 | 1 | 2 (25.00) |
| Complex Cardiac Defects | 4 | 13 | 13 | 0 | 1 | 90.68  (65.13 – 99.99) | 99.92  (99.75 – 99.99) | 0 | 1 | 0 (0.00) |

*Rates for individual anomalies were only calculated for those conditions where more than 10 cases were reported (see methods). This excluded the following abnormalities: Cardiomegaly (n=7), Truncus Arteriosus (n=8), Tricuspid Valve Atresia (n=7), Cardiomyopathy (n=6), Ebstein Anomaly (n=6), Aortic Arch Hypoplasia (n=5), Aortic Stenosis (n=5), Ectopia Cordis (n=4), Double Inlet Left Ventricle (n=2), Hypoplastic Right Heart Syndrome (n=2), Interrupted Aortic Arch (n=2), Mitral Valve Atresia (n=2), Univentricle (n=2), Endocardial Fibroelastosis (n=1), Interrupted IVC (N=1), Pentalogy of Cantrell (n=1), Pulmonary Valve Regurgitation (n=1), Total Anomalous Pulmonary Venous Drainage (n=1). Detailed data available on request.

Anomalies detected (TP) refers to anomalies which were either correctly diagnosed, suspected or identified as an anatomical anomaly of unknown significance at the first trimester ultrasound assessment. FP refers to anomalies which were diagnosed, suspected or identified as an anatomical anomaly of unknown significance in the first trimester, but where subsequent assessment of the fetal heart showed no cardiac abnormality. Diagnosis change refers to anomalies which were diagnosed or suspected in the first trimester and where the specific nature of the cardiac anomaly was found to be different at a later gestational age (see supplemental table 14).

**Table S18** Comparison of detection rates for individual cardiac anomalies in non-high-risk and high-risk populations

| **Anomaly** | **Detection rate for individual cardiac anomalies**  **% (95% C.I.)** | |
| --- | --- | --- |
|  | **Non-High Risk** | **High Risk** |
| HLHS | 73.28  (59.86 – 84.82) | 69.29  (43.68 – 89.75) |
| HRHS | 91.65  (77.23 – 99.21) | - |
| Univentricle | 71.21  (52.11 – 87.03) | - |
| TOF | 40.95  (30.16 – 52.20) | 53.66  (32.89 – 73.78) |
| TGA | 45.05  (29.29 – 61.35) | 81.48  (62.56 – 94.74) |
| CoA | 37.23  (23.96 – 51.56) | 59.88  (43.30 – 75.39) |
| VSD | 23.92  (14.41 – 34.97) | 47.50  (26.75 – 68.70) |
| AVSD | 77.24  (63.62 – 88.42) | 83.62  (69.56 – 93.93) |
| ASD | 21.53  (6.78 – 41.66) | 16.11  (2.40 – 38.60) |
| Truncus Arteriosus | 76.73  (58.94 – 90.62) | - |
| DORV | 63.11  (44.90 – 79.59) | 56.99  (27.99 – 83.58) |
| Heterotaxy Syndromes | 72.59  (55.75 – 86.63) | 83.05  (64.00 – 95.85) |
| Ectopia Cordis | 93.26  (76.03 – 99.98) | - |
| Ebstein’s Anomaly | 25.03  (4.83 – 54.08) | - |
| Rhabdo-myoma | 4.87  (0.19 – 22.09) | - |
| Aortic Stenosis | 38.81  (15.77 – 64.90) | - |
| Pulmonary valve/  Pulmonary Artery Stenosis | 19.45  (8.99 – 32.74) | 29.12  (7.50 – 57.68) |
| Pulmonary Atresia | 59.68  (23.63 – 90.53) | 65.26  (38.77 – 87.45) |
| Tricuspid Atresia/  Dysplasia | 88.63  (76.00 – 96.94) | - |
| Complex Cardiac Defects | 76.31  (57.46 – 90.92) | 90.68  (65.13 – 99.99) |
